# Supplementary material for: Latent Classes of Adverse and Benevolent Childhood Experiences in a Multinational Sample of Parents and Their Relation to Parent, Child, and Family Functioning during the COVID-19 Pandemic
Source: Int J Environ Res Public Health. 2022 Oct 20;19(20):13581. doi: 10.3390/ijerph192013581 (PMC9603677; doi:10.3390/ijerph192013581)
Supplement: Supplementary file 1 [file ijerph-19-13581-s001.zip › ijerph-1922698-supplementary.pdf]

Supplementary Information

TITLE: Latent classes of adverse and benevolent childhood experiences in a multinational sample of parents and their relation to parent, child, and family functioning during the COVID-19 pandemic

AUTHORS: Dylan Johnson[1], Dillon T. Browne[2], Robert D. Meade[3,4], Heather Prime[5], Mark Wade[1]

AFFILIATIONS:

1 Department of Applied Psychology and Human Development, University of Toronto, Toronto, Ontario, Canada

2 Centre for Mental Health Research and Treatment, Department of Psychology, University of Waterloo, Waterloo, Ontario, Canada

3 Human and Environmental Physiology Research Unit, School of Human Kinetics, University of Ottawa, Ottawa, Ontario, Canada

4 Harvard T.H. Chan School of Public Health, Harvard University, Boston, MA, USA

5 Department of Psychology, York University, Toronto, Ontario, Canada

Table of Contents

Table S1. Indicator items used in latent class analysis 6

Table S2. Latent class analysis of adverse childhood experiences and benevolent childhood experiences model fit indices 7

Figure S1. Distributional assumptions in the association between adversity and benevolence latent class and anxiety, adjusted for age, sex, socioeconomic status, ethnicity, immigration status, and single-parent status 9

Figure S2. Distributional assumptions in the association between adversity and benevolence latent class and distress, adjusted for age, sex, socioeconomic status, ethnicity, immigration status, and single-parent status 10

Figure S3. Distributional assumptions in the association between adversity and benevolence latent class and posttraumatic stress, adjusted for age, sex, socioeconomic status, ethnicity, immigration status, and single-parent status 11

Figure S4. Distributional assumptions in the association between adversity and benevolence latent class and substance use, adjusted for age, sex, socioeconomic status, ethnicity, immigration status, and single-parent status 12

Figure S5. Distributional assumptions in the association between adversity and benevolence latent class and family dysfunction, adjusted for age, sex, socioeconomic status, ethnicity, immigration status, and single-parent status 13

Figure S6. Correlational matrix for parent outcomes of anxiety, distress, posttraumatic stress, substance use, and family dysfunction 14

Table S3. Multiple linear regression model in the association between adversity and benevolence latent class and parent anxiety 15

Table S4. Multiple linear regression model in the association between adversity and benevolence latent class and parent distress 15

Table S5. Multiple linear regression model in the association between adversity and benevolence latent class and parent posttraumatic stress 16

Table S6. Multiple linear regression model in the association between adversity and benevolence latent class and parent substance use 16

Table S7. Multiple linear regression model in the association between adversity and benevolence latent class and family dysfunction 17

Figure S7. Distributional assumptions in the association between adversity and benevolence latent class and older-child anger, adjusted for age, sex, socioeconomic status, ethnicity, immigration status, and single-parent status 19

Figure S8. Distributional assumptions in the association between adversity and benevolence latent class and younger-born-child anger, adjusted for age, sex, socioeconomic status, ethnicity, immigration status, and single parent status 20

Figure S9. Distributional assumptions in the association between adversity and benevolence latent class and averaged older-and-younger-child anger, adjusted for age, sex, socioeconomic status, ethnicity, immigration status, and single parent status 21

Figure S10. Distributional assumptions in the association between adversity and benevolence latent class and older-child anxiety, adjusted for age, sex, socioeconomic status, ethnicity, immigration status, and single parent status 22

Figure S11. Distributional assumptions in the association between adversity and benevolence latent class and younger-born-child anxiety, adjusted for age, sex, socioeconomic status, ethnicity, immigration status, and single parent status 23

Figure S12. Distributional assumptions in the association between adversity and benevolence latent class and averaged older-and-younger-child anxiety, adjusted for age, sex, socioeconomic status, ethnicity, immigration status, and single parent status 24

Figure S13. Distributional assumptions in the association between adversity and benevolence latent class and older-born-child depression, adjusted for age, sex, socioeconomic status, ethnicity, immigration status, and single parent status 25

Figure S14. Distributional assumptions in the association between adversity and benevolence latent class and younger-born-child depression, adjusted for age, sex, socioeconomic status, ethnicity, immigration status, and single parent status 26

Figure S15. Distributional assumptions in the association between adversity and benevolence latent class and averaged older-and-younger-child depression, adjusted for age, sex, socioeconomic status, ethnicity, immigration status, and single parent status 27

Figure S16. Distributional assumptions in the association between adversity and benevolence latent class and older-born-child positive coping, adjusted for age, sex, socioeconomic status, ethnicity, immigration status, and single parent status 28

Figure S17. Distributional assumptions in the association between adversity and benevolence latent class and younger-born-child positive coping, adjusted for age, sex, socioeconomic status, ethnicity, immigration status, and single parent status 29

Figure S18. Distributional assumptions in the association between adversity and benevolence latent class and averaged older-and-younger-child positive coping, adjusted for age, sex, socioeconomic status, ethnicity, immigration status, and single parent status 30

Figure S19. Distributional assumptions in the association between adversity and benevolence latent class and older-born-child reported parenting quality, adjusted for age, sex, socioeconomic status, ethnicity, immigration status, and single parent status 31

Figure S20. Distributional assumptions in the association between adversity and benevolence latent class and younger-born-child reported parenting quality, adjusted for age, sex, socioeconomic status, ethnicity, immigration status, and single parent status 32

Figure S21. Distributional assumptions in the association between adversity and benevolence latent class and averaged older-and-younger-child reported parenting quality, adjusted for age, sex, socioeconomic status, ethnicity, immigration status, and single parent status 33

Figure S22. Correlational matrix for older-born-child outcomes of anger, anxiety, depression, positive coping, and parenting quality 34

Figure S23. Correlational matrix for younger-born-child outcomes of anger, anxiety, depression, positive coping, and parenting quality 35

Figure S24. Correlational matrix for averaged older-and-younger-child outcomes of anger, anxiety, depression, positive coping, and parenting quality 36

Table S8. Distribution of younger child mental health, positive coping, and parenting quality, among latent classes of adversity and benevolence. 37

Table S9. Multiple linear regression model in the association between adversity and benevolence latent class and older-born-child anger 38

Table S10. Multiple linear regression model in the association between adversity and benevolence latent class and younger-born-child anger 38

Table S11. Multiple linear regression model in the association between adversity and benevolence latent class and averaged older and younger child anger 39

Table S12. Multiple linear regression model in the association between adversity and benevolence latent class and older-child anxiety 39

Table S13. Multiple linear regression model in the association between adversity and benevolence latent class and younger-born-child anxiety 40

Table S14. Multiple linear regression model in the association between adversity and benevolence latent class and averaged older and younger child anxiety 40

Table S15. Multiple linear regression model in the association between adversity and benevolence latent class and older-child depression 41

Table S16. Multiple linear regression model in the association between adversity and benevolence latent class and younger-child depression 41

Table S17. Multiple linear regression model in the association between adversity and benevolence latent class and averaged older and younger child depression 42

Table S18. Multiple linear regression model in the association between adversity and benevolence latent class and older-child 42

Table S19. Multiple linear regression model in the association between adversity and benevolence latent class and younger-child 43

Table S20. Multiple linear regression model in the association between adversity and benevolence latent class and averaged older and younger child 43

Table S21. Multiple linear regression model in the association between adversity and benevolence latent class and older-child parenting quality 44

Table S22. Multiple linear regression model in the association between adversity and benevolence latent class and younger-child parenting quality 44

Table S23. Multiple linear regression model in the association between adversity and benevolence latent class and averaged older and younger child parenting quality 45

Table S24. Frequency of missing data per variable 46

Table S25. R Packages used for analyses and data presentation in R Studio (version 4.0.2) 47

Table S1. Indicator items used in latent class analysis

Adverse Childhood Experiences (ACEs) Items

1. *Did a parent or other adult in the household* ***often or very often:*** *Swear at you, insult you, put you down, or humiliate you?* **or** *Act in a way that made you afraid that you might be physically hurt?*
2. *Did a parent or other adult in the household* ***often or very often:*** *Push, grab, slap, or throw something at you?* ***or ever*** *hit you so hard that you had marks or were injured?*
3. *Did an adult or person at least 5 years older than you* ***ever:*** *Touch or fondle you or have you touch their body in a sexual way?* ***or*** *Attempt or actually have oral, anal, or vaginal intercourse with you?*
4. *Did you* ***often or very often*** *feel that: No one in your family loved you or thought you were important or special?* ***or*** *Your family didn't look out for each other, feel close to each other, or support each other?*
5. *Did you* ***often or very often*** *feel that: You didn't have enough to eat, had to wear dirty clothes, and had no one to protect you?* ***or*** *Your parents were too drunk or high to take care of you or take you to the doctor if you needed it?*
6. *Was a biological parent* ***ever*** *los t to you through divorce, abandonment, or other reason?*
7. *Was your mother or stepmother:* ***Often or very often*** *pushed, grabbed, slapped, or had something thrown at her?* ***or sometimes, often, or very often*** *kicked, bitten, hit with a fist, or hit with something hard?* ***or ever*** *repeatedly hit over at least a few minutes or threatened with a gun or knife?*
8. *Did you live with anyone who was a problem drinker or alcoholic, or who used street drugs?*
9. *Was a household member depressed or mentally ill, or did a household member attempt suicide?*
10. *Did a household member go to prison?*
11. *Did other kids, including brothers or sisters,* ***often or very often*** *hit you, threaten you, pick on you or insult you?*
12. *Did you* ***often or very often*** *feel lonely, rejected or that nobody liked you?*
13. *Did you live for 2 or more years in a neighborhood that was dangerous, or where you saw people being assaulted?*
14. *Was there a period of 2 or more years when your family was very poor or on public assistance?*

Benevolent Childhood Experiences (BCEs) Items

1. *Did you have at least one caregiver with whom you felt safe?*
2. *Did you have at least one good friend?*
3. *Did you have beliefs that gave you comfort?*
4. *Did you like school?*
5. *Did you have at least one teacher who cared about you?*
6. *Did you have good neighbors?*
7. *Was there an adult (not a parent/caregiver or the person from #1) who could provide*

Table S2. Latent class analysis of adverse childhood experiences and benevolent childhood experiences model fit indices

Model	BLRT	BIC	Entropy	Smallest profile
1 class	--	10782.462	--	--
2 class	p<0.001	9371.184	0.90	34.37%
3 class	p<0.001	9253.715	0.87	9.69%
4 class	**p<0.001**	**9232.937**	**0.86**	**11.15%**
5 class	p=0.0986	9276.008	0.88	5.30%%
6 class	Model failed to converge	--	--	--

BIC: Bayesian information criterion
BLRT: Bootstrapped likelihood-ratio testing

Parent Outcomes

Figure S1. Distributional assumptions in the association between adversity and benevolence latent class and anxiety, adjusted for age, sex, socioeconomic status, ethnicity, immigration status, and single-parent status

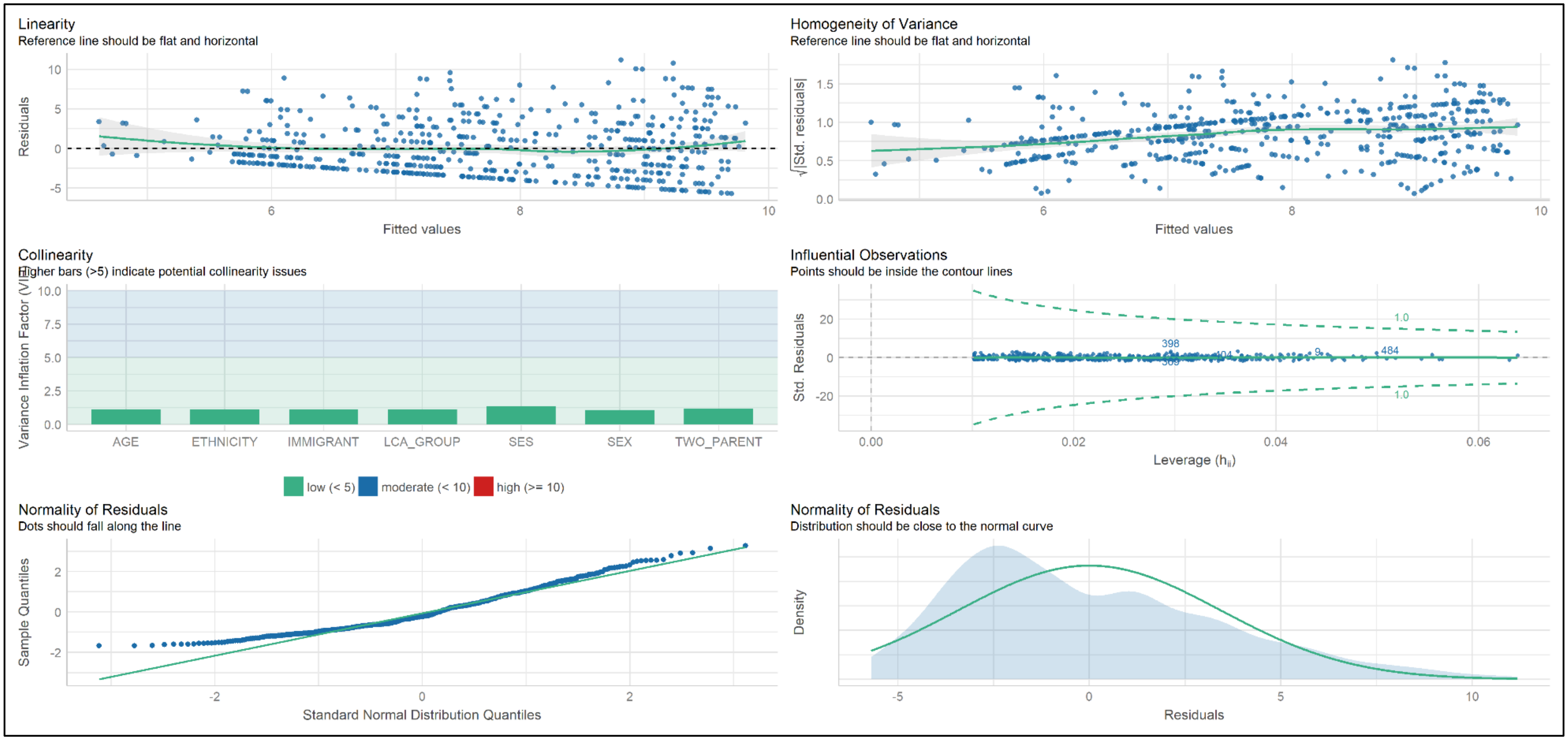

Missing covariate data were estimated using multiple imputation via chained equations. Data were analyzed with multivariable linear regression. Standard errors were corrected for using an HC3 adjustment. Analysis was run on 10 imputed data sets and the outcomes were combined using Rubin's Rules. The first imputed data set was used to display distributional assumptions depicted above.

Figure S2. Distributional assumptions in the association between adversity and benevolence latent class and distress, adjusted for age, sex, socioeconomic status, ethnicity, immigration status, and single-parent status

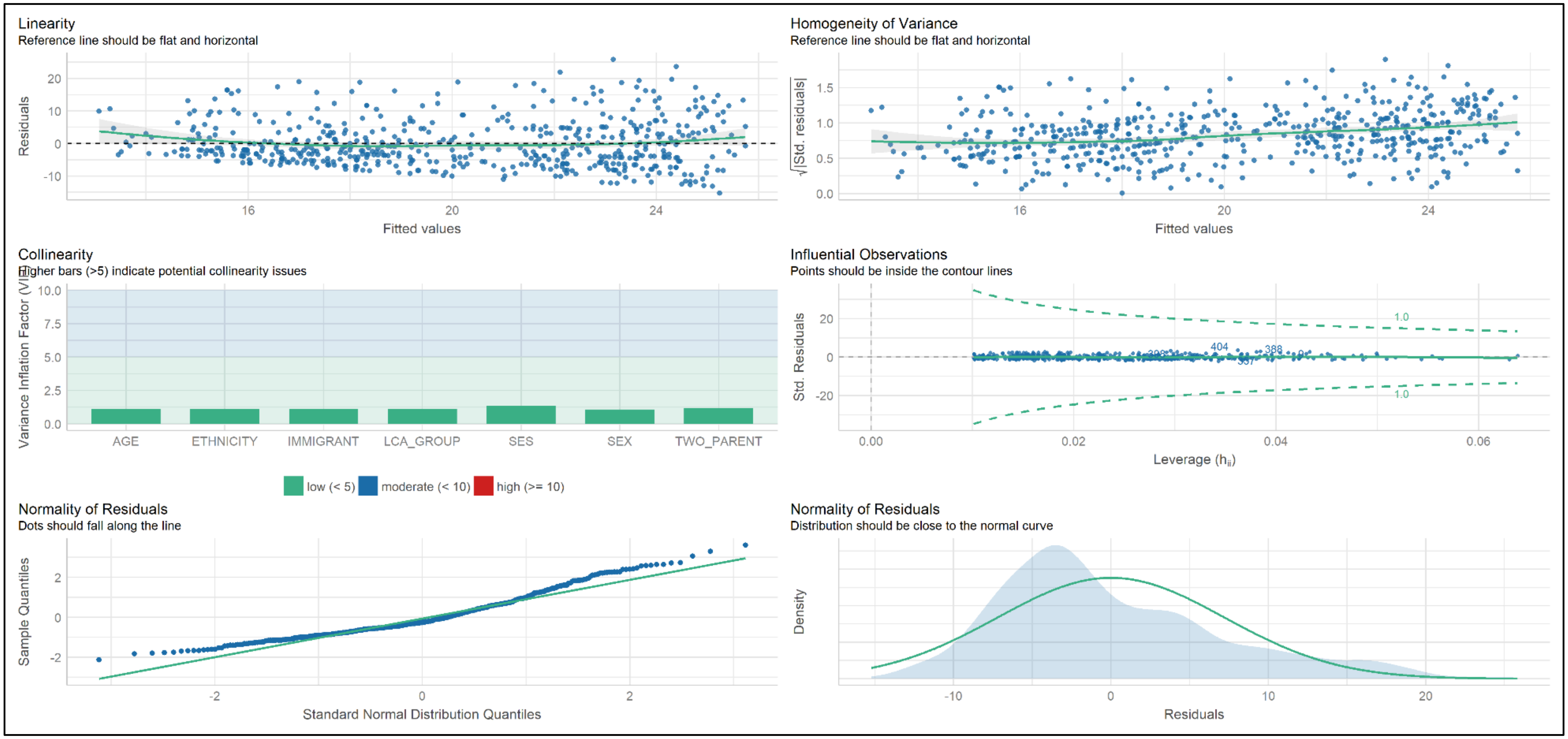

Missing covariate data were estimated using multiple imputation via chained equations. Data were analyzed with multivariable linear regression. Standard errors were corrected for using an HC3 adjustment. Analysis was run on 10 imputed data sets and the outcomes were combined using Rubin's Rules. The first imputed data set was used to display distributional assumptions depicted above.

Figure S3. Distributional assumptions in the association between adversity and benevolence latent class and posttraumatic stress, adjusted for age, sex, socioeconomic status, ethnicity, immigration status, and single-parent status

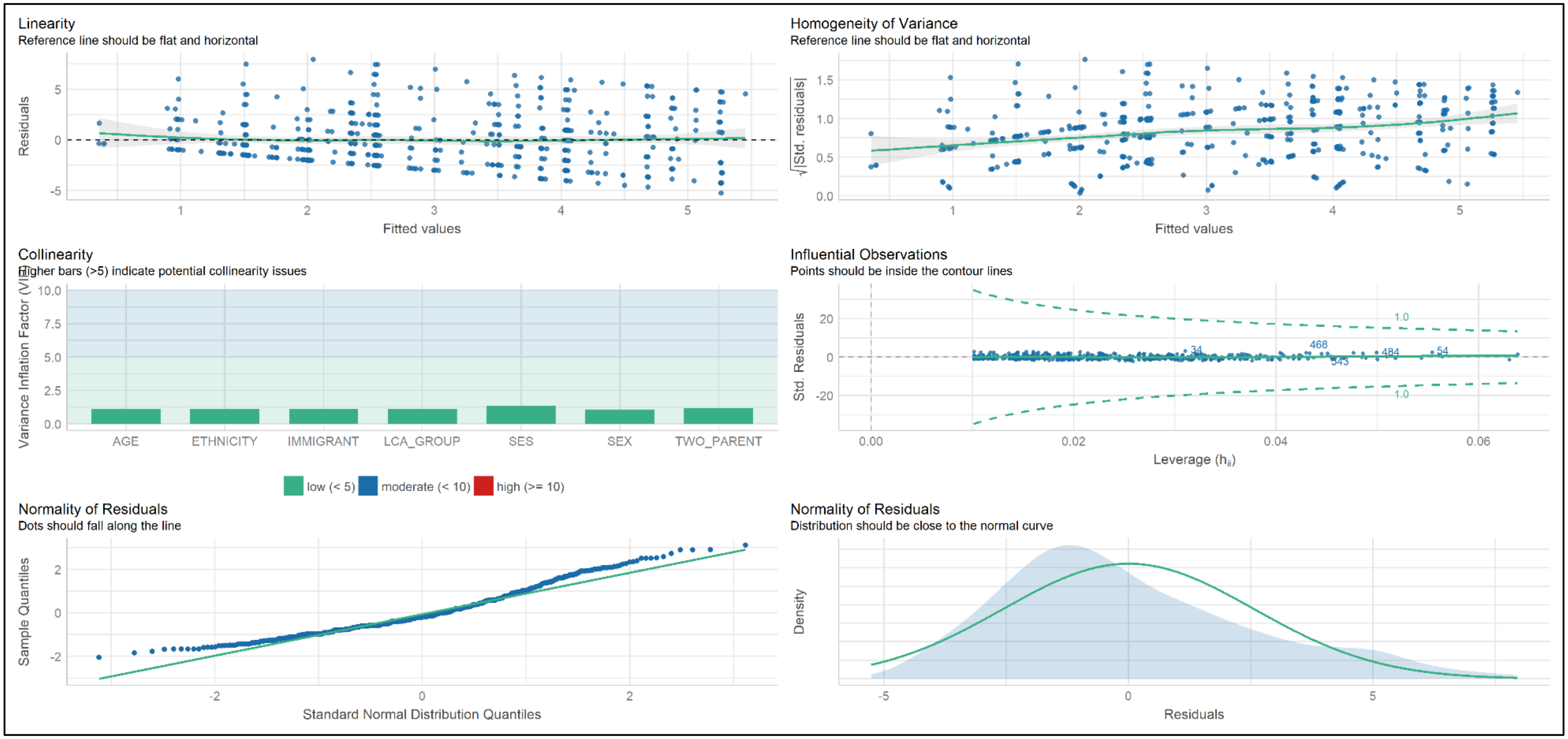

Missing covariate data were estimated using multiple imputation via chained equations. Data were analyzed with multivariable linear regression. Standard errors were corrected for using an HC3 adjustment. Analysis was run on 10 imputed data sets and the outcomes were combined using Rubin's Rules. The first imputed data set was used to display distributional assumptions depicted above.

Figure S4. Distributional assumptions in the association between adversity and benevolence latent class and substance use, adjusted for age, sex, socioeconomic status, ethnicity, immigration status, and single-parent status

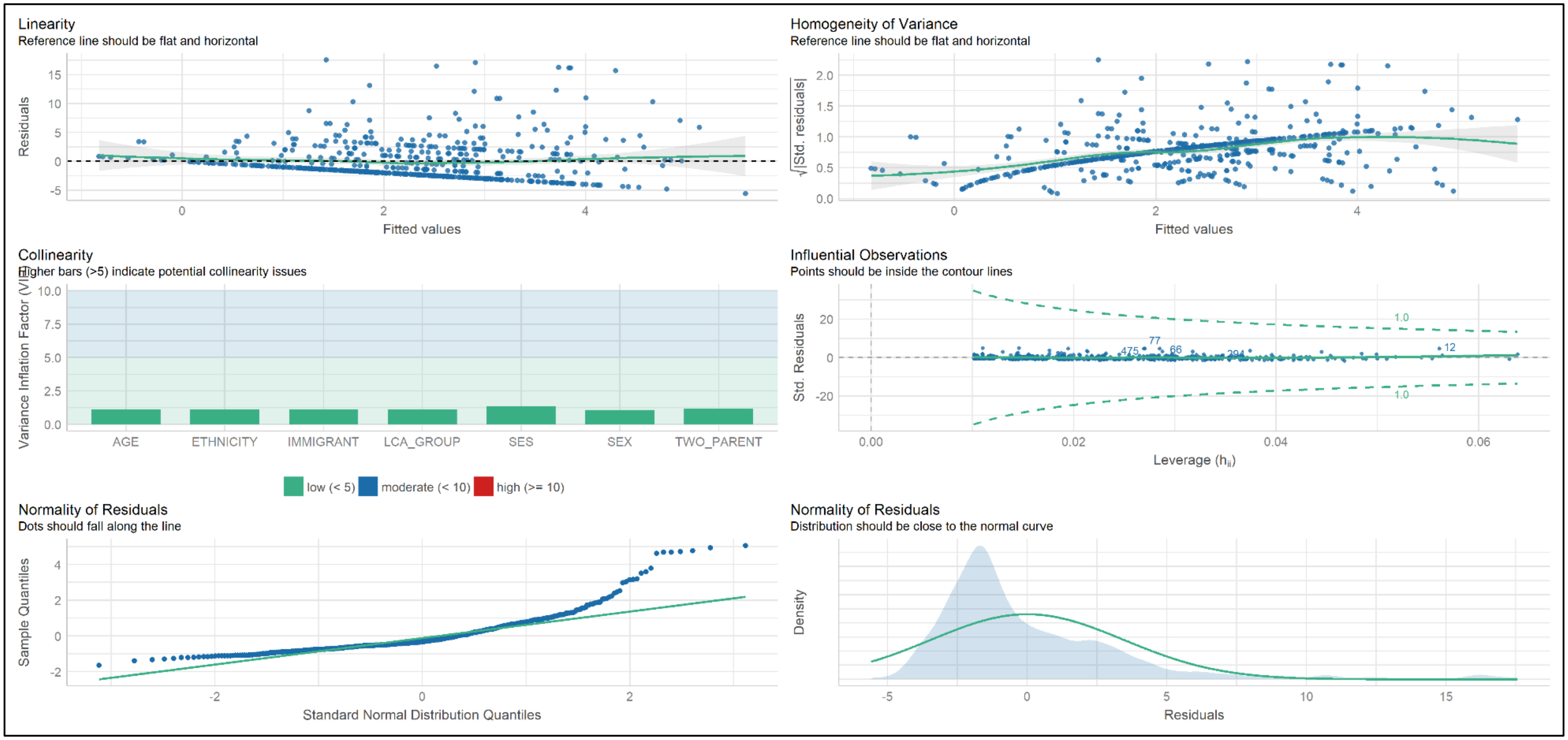

Missing covariate data were estimated using multiple imputation via chained equations. Data were analyzed with multivariable linear regression. Standard errors were corrected for using an HC3 adjustment. Analysis was run on 10 imputed data sets and the outcomes were combined using Rubin’s Rules. The first imputed data set was used to display distributional assumptions depicted above.

Figure S5. Distributional assumptions in the association between adversity and benevolence latent class and family dysfunction, adjusted for age, sex, socioeconomic status, ethnicity, immigration status, and single-parent status

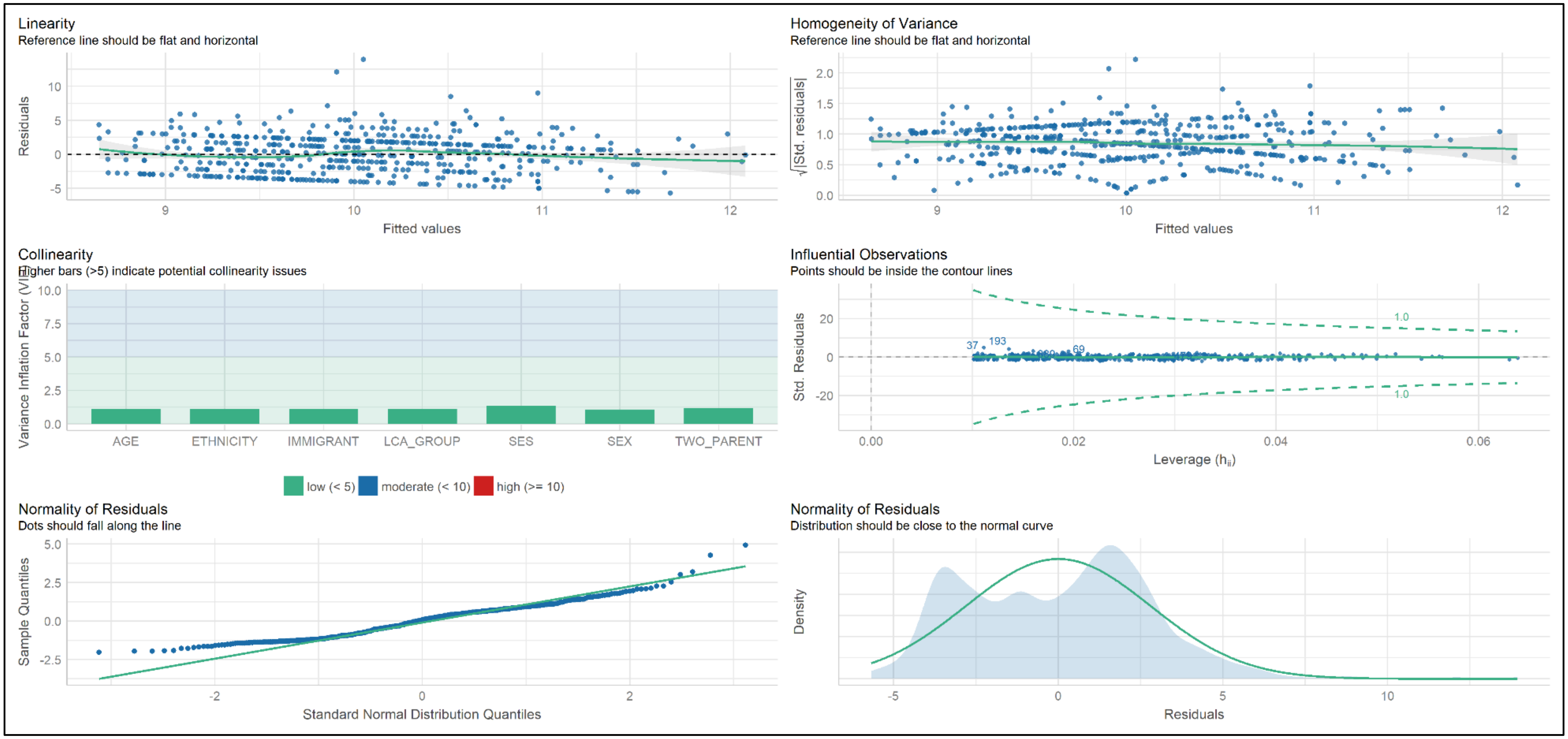

Missing covariate data were estimated using multiple imputation via chained equations. Data were analyzed with multivariable linear regression. Standard errors were corrected for using an HC3 adjustment. Analysis was run on 10 imputed data sets and the outcomes were combined using Rubin's Rules. The first imputed data set was used to display distributional assumptions depicted above.

Figure S6. Correlational matrix for parent outcomes of anxiety, distress, posttraumatic stress, substance use, and family dysfunction

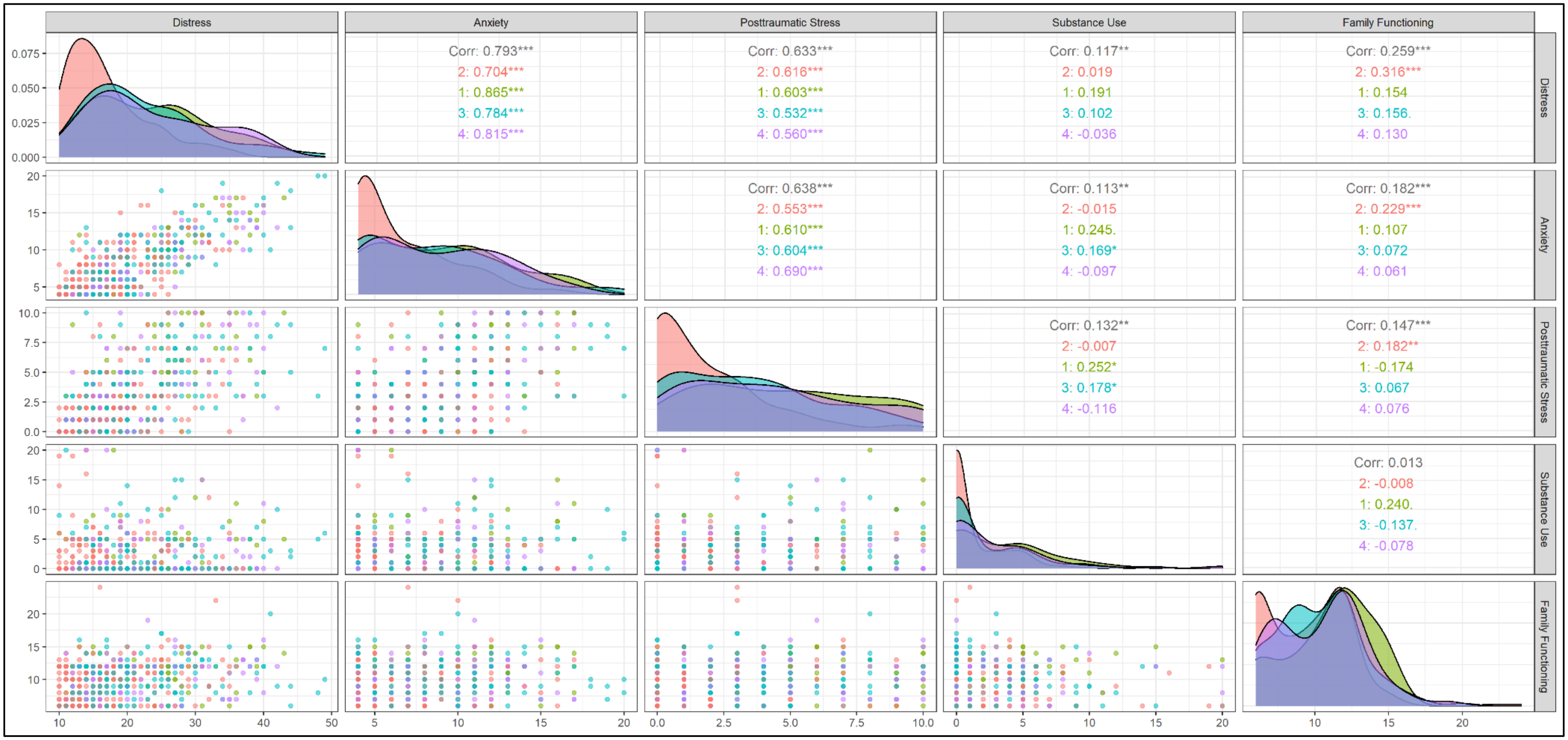

Table S3. Multiple linear regression model in the association between adversity and benevolence latent class and parent anxiety (n=545)

Pairwise Comparison		Unadjusted			Adjusted†		
Reference	Comparator	Mean Difference	Standard Error	t-statistic	Mean Difference	Standard Error	t-statistic
High ACE/Mod BCE	Low ACE/High BCE	-2.39	0.54	-4.43***	-2.08	0.54	-3.82***
High ACE/Mod BCE	Mod ACE/High BCE	-0.51	0.61	-0.84	-0.28	0.62	-0.45
High ACE/Mod BCE	Mod ACE/Low BCE	-0.23	0.68	-0.34	-0.19	0.67	-0.28
Low ACE/High BCE	Mod ACE/High BCE	1.88	0.38	4.92***	1.80	0.38	4.68***
Low ACE/High BCE	Mod ACE/Low BCE	2.16	0.49	4.42***	1.89	0.48	3.91***
Mod ACE/High BCE	Mod ACE/Low BCE	0.29	0.56	0.51	0.09	0.56	0.16

†Adjusted for age, sex, socioeconomic status, ethnicity, immigration status, and one-parent household status
*p<0.05
**p<0.01
***p<0.001

Table S4. Multiple linear regression model in the association between adversity and benevolence latent class and parent distress (n=546)

Pairwise Comparison		Unadjusted			Adjusted†		
Reference	Comparator	Mean Difference	Standard Error	t-statistic	Mean Difference	Standard Error	t-statistic
High ACE/Mod BCE	Low ACE/High BCE	-6.23	1.14	-5.44***	-5.44	1.15	-4.74***
High ACE/Mod BCE	Mod ACE/High BCE	-0.85	1.29	-0.66	-0.34	1.30	-0.26
High ACE/Mod BCE	Mod ACE/Low BCE	0.28	1.51	0.19	0.37	1.48	0.25
Low ACE/High BCE	Mod ACE/High BCE	5.38	0.79	6.81***	5.11	0.79	6.44***
Low ACE/High BCE	Mod ACE/Low BCE	6.51	1.11	5.85***	5.82	1.09	5.32***
Mod ACE/High BCE	Mod ACE/Low BCE	1.13	1.26	0.90	0.71	1.24	0.57

†Adjusted for age, sex, socioeconomic status, ethnicity, immigration status, and one-parent household status
*p<0.05
**p<0.01
***p<0.001

Table S5. Multiple linear regression model in the association between adversity and benevolence latent class and parent posttraumatic stress (n=545)

Pairwise Comparison		Unadjusted			Adjusted†		
Reference	Comparator	Mean Difference	Standard Error	t-statistic	Mean Difference	Standard Error	t-statistic
High ACE/Mod BCE	Low ACE/High BCE	-2.91	0.43	-6.78***	-2.72	0.43	-6.26***
High ACE/Mod BCE	Mod ACE/High BCE	-1.35	0.47	-2.89**	-1.21	0.47	-2.58*
High ACE/Mod BCE	Mod ACE/Low BCE	-0.48	0.55	-0.87	-0.38	0.55	-0.69
Low ACE/High BCE	Mod ACE/High BCE	1.56	0.27	5.80***	1.51	0.27	5.59***
Low ACE/High BCE	Mod ACE/Low BCE	2.44	0.40	6.09***	2.34	0.40	5.83***
Mod ACE/High BCE	Mod ACE/Low BCE	0.87	0.44	1.98	0.83	0.44	1.87

†Adjusted for age, sex, socioeconomic status, ethnicity, immigration status, and one-parent household status
*p<0.05
**p<0.01
***p<0.001

Table S6. Multiple linear regression model in the association between adversity and benevolence latent class and parent substance use (n=545)

Pairwise Comparison		Unadjusted			Adjusted†		
Reference	Comparator	Mean Difference	Standard Error	t-statistic	Mean Difference	Standard Error	t-statistic
High ACE/Mod BCE	Low ACE/High BCE	-1.94	0.58	-3.38***	-1.71	0.55	-3.09**
High ACE/Mod BCE	Mod ACE/High BCE	-1.17	0.62	-1.88	-0.86	0.61	-1.41
High ACE/Mod BCE	Mod ACE/Low BCE	-0.68	0.75	-0.9	-0.61	0.74	-0.83
Low ACE/High BCE	Mod ACE/High BCE	0.77	0.36	2.14*	0.85	0.36	2.36*
Low ACE/High BCE	Mod ACE/Low BCE	1.27	0.55	2.29*	1.10	0.57	1.93
Mod ACE/High BCE	Mod ACE/Low BCE	0.49	0.6	0.82	0.26	0.61	0.42

†Adjusted for age, sex, socioeconomic status, ethnicity, immigration status, and one-parent household status
*p<0.05
**p<0.01
***p<0.001

Table S7. Multiple linear regression model in the association between adversity and benevolence latent class and family dysfunction (n=547)

Pairwise Comparison		Unadjusted			Adjusted†		
Reference	Comparator	Mean Difference	Standard Error	t-statistic	Mean Difference	Standard Error	t-statistic
High ACE/Mod BCE	Low ACE/High BCE	-1.57	0.40	-3.87***	-1.59	0.43	-3.72***
High ACE/Mod BCE	Mod ACE/High BCE	-0.81	0.43	-1.87	-0.81	0.45	-1.81
High ACE/Mod BCE	Mod ACE/Low BCE	-0.70	0.50	-1.38	-0.79	0.52	-1.51
Low ACE/High BCE	Mod ACE/High BCE	0.76	0.29	2.62**	0.77	0.29	2.63**
Low ACE/High BCE	Mod ACE/Low BCE	0.87	0.39	2.23*	0.80	0.41	1.97*
Mod ACE/High BCE	Mod ACE/Low BCE	0.11	0.42	0.27	0.02	0.44	0.06

†Adjusted for age, sex, socioeconomic status, ethnicity, immigration status, and one-parent household status
*p<0.05
**p<0.01
***p<0.001

Child Outcomes

Figure S7. Distributional assumptions in the association between adversity and benevolence latent class and older-child anger, adjusted for age, sex, socioeconomic status, ethnicity, immigration status, and single-parent status

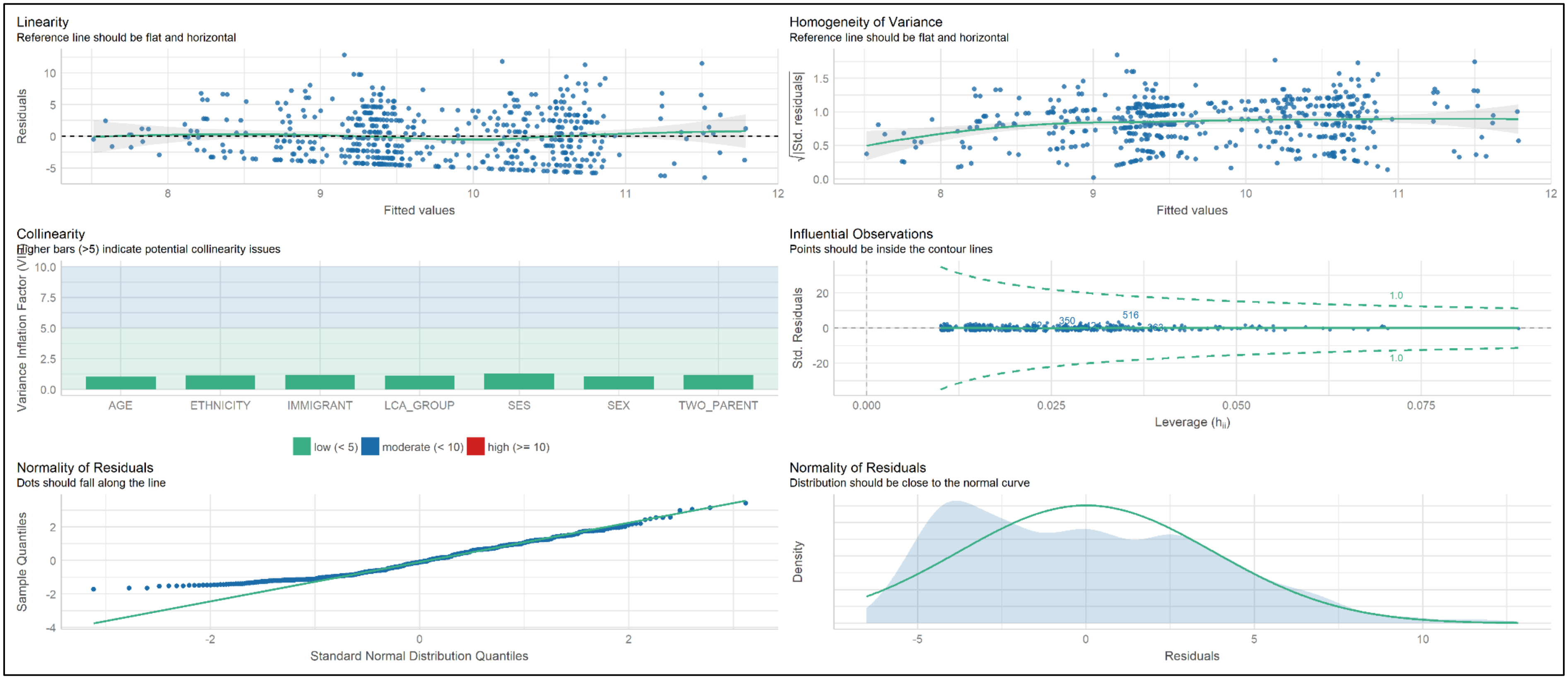

Missing covariate data were estimated using multiple imputation via chained equations. Data were analyzed with multivariable linear regression. Standard errors were corrected for using an HC3 adjustment. Analysis was run on 10 imputed data sets and the outcomes were combined using Rubin's Rules. The first imputed data set was used to display distributional assumptions depicted above.

Figure S8. Distributional assumptions in the association between adversity and benevolence latent class and younger-born-child anger, adjusted for age, sex, socioeconomic status, ethnicity, immigration status, and single parent status

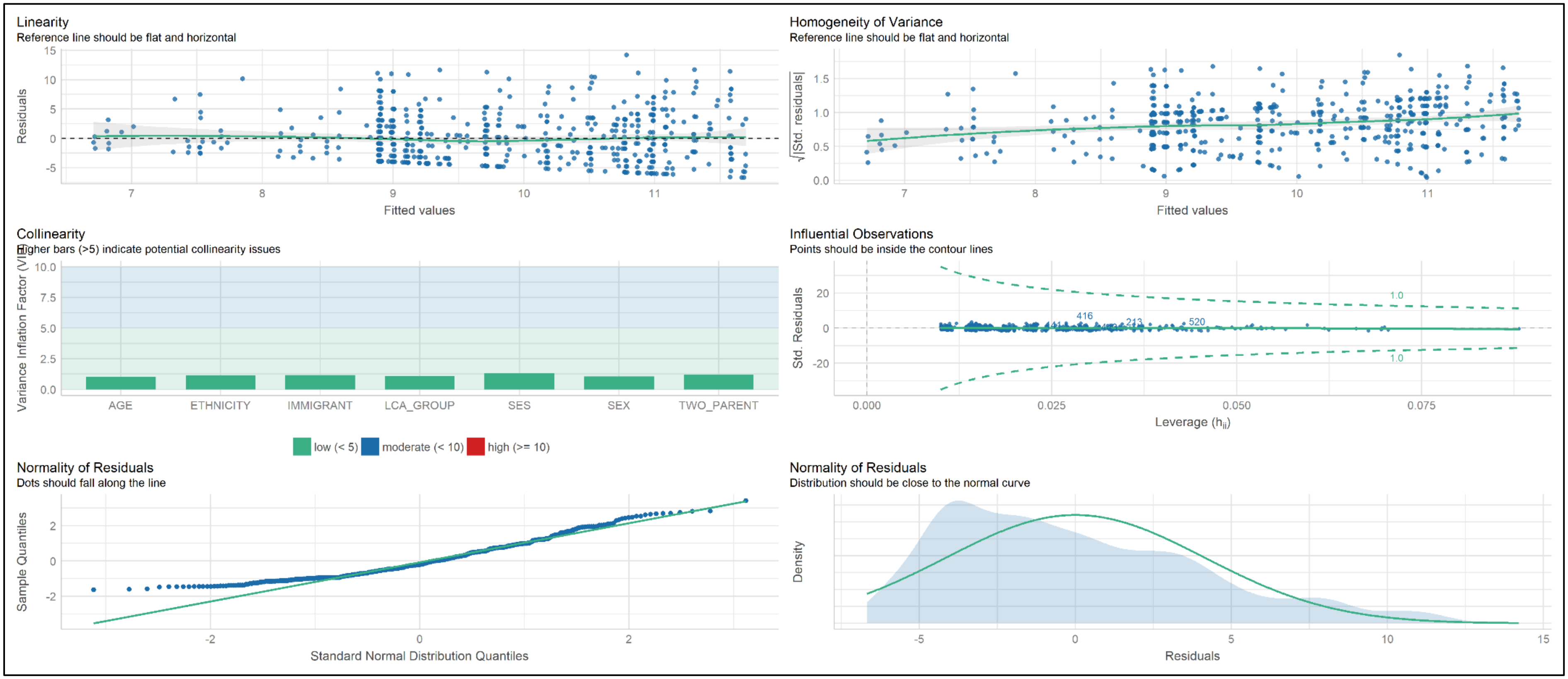

Missing covariate data were estimated using multiple imputation via chained equations. Data were analyzed with multivariable linear regression. Standard errors were corrected for using an HC3 adjustment. Analysis was run on 10 imputed data sets and the outcomes were combined using Rubin’s Rules. The first imputed data set was used to display distributional assumptions depicted above.

Figure S9. Distributional assumptions in the association between adversity and benevolence latent class and averaged older-and-younger-child anger, adjusted for age, sex, socioeconomic status, ethnicity, immigration status, and single parent status

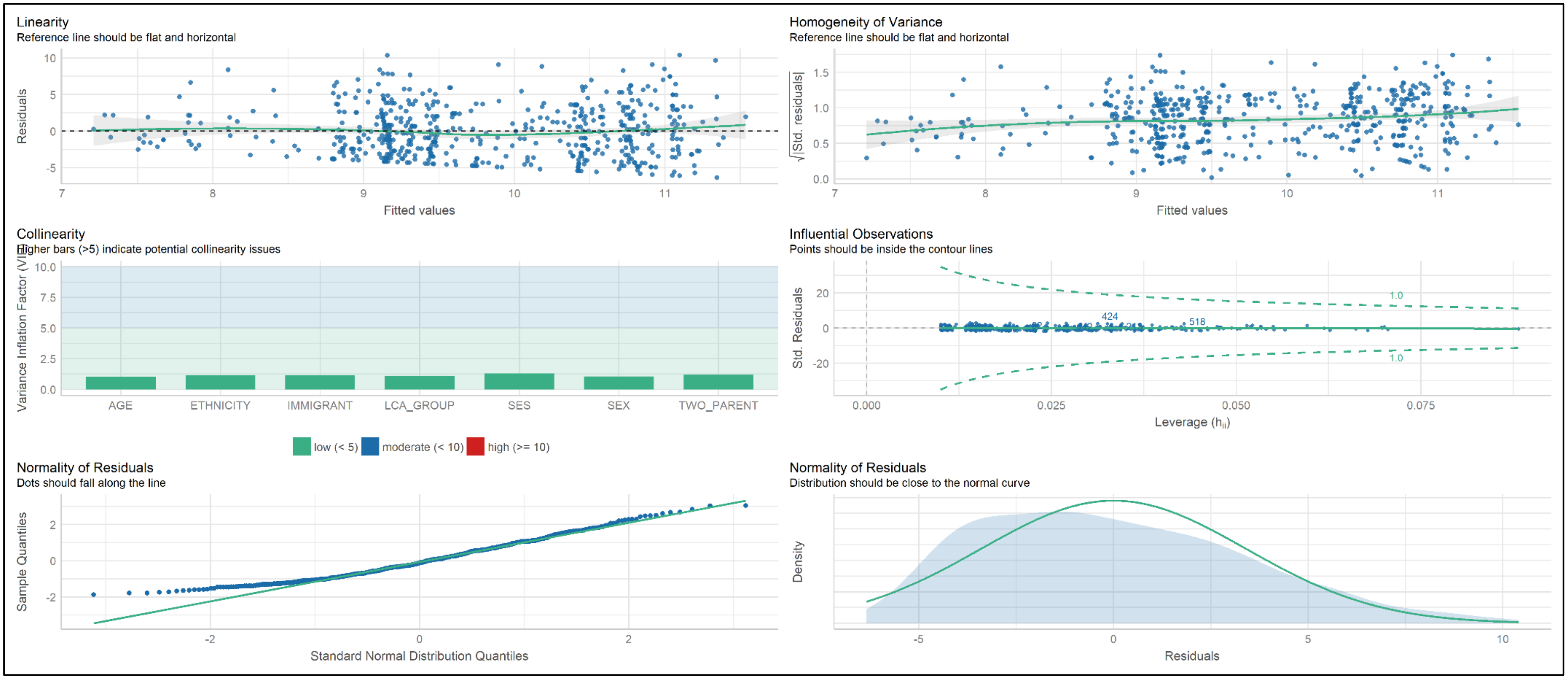

Missing covariate data were estimated using multiple imputation via chained equations. Data were analyzed with multivariable linear regression. Standard errors were corrected for using an HC3 adjustment. Analysis was run on 10 imputed data sets and the outcomes were combined using Rubin's Rules. The first imputed data set was used to display distributional assumptions depicted above.

Figure S10. Distributional assumptions in the association between adversity and benevolence latent class and older-child anxiety, adjusted for age, sex, socioeconomic status, ethnicity, immigration status, and single parent status

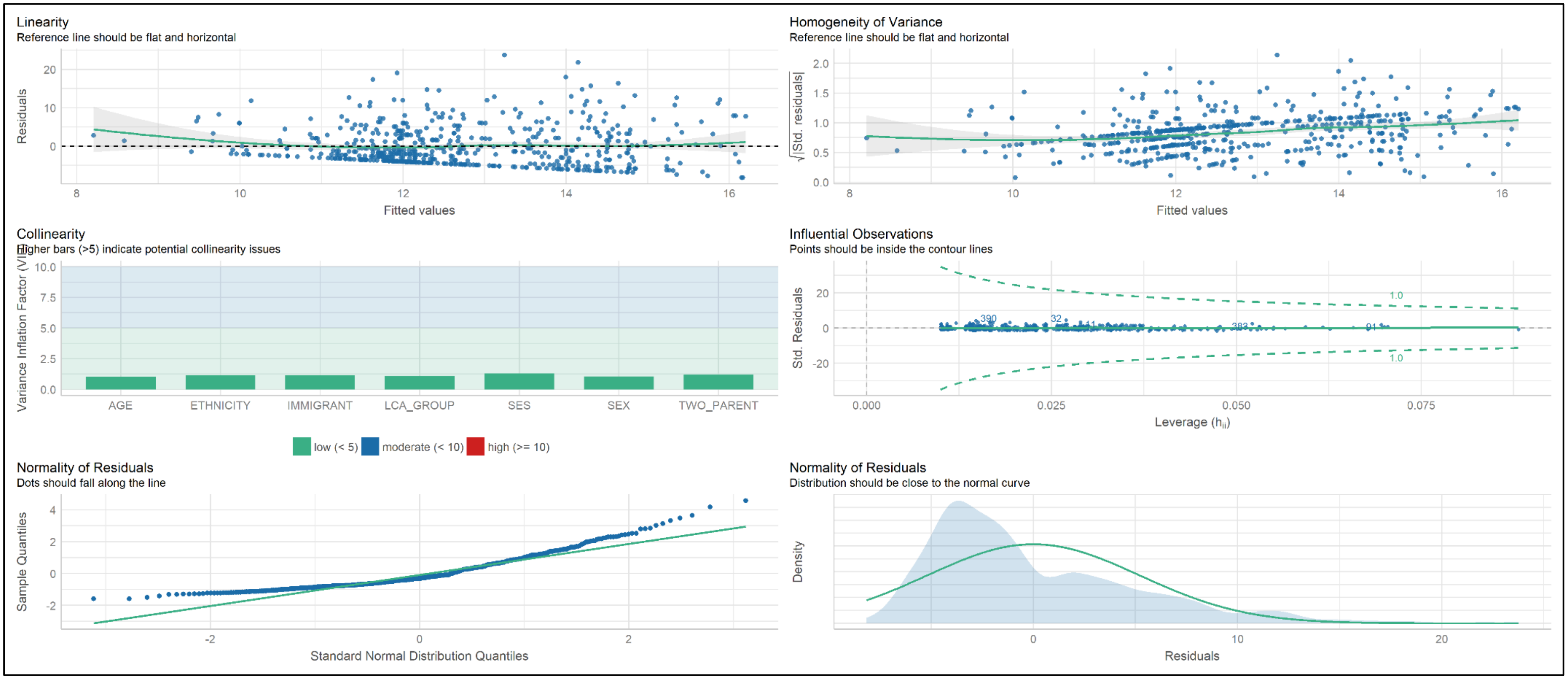

Missing covariate data were estimated using multiple imputation via chained equations. Data were analyzed with multivariable linear regression. Standard errors were corrected for using an HC3 adjustment. Analysis was run on 10 imputed data sets and the outcomes were combined using Rubin's Rules. The first imputed data set was used to display distributional assumptions depicted above.

Figure S11. Distributional assumptions in the association between adversity and benevolence latent class and younger-born-child anxiety, adjusted for age, sex, socioeconomic status, ethnicity, immigration status, and single parent status

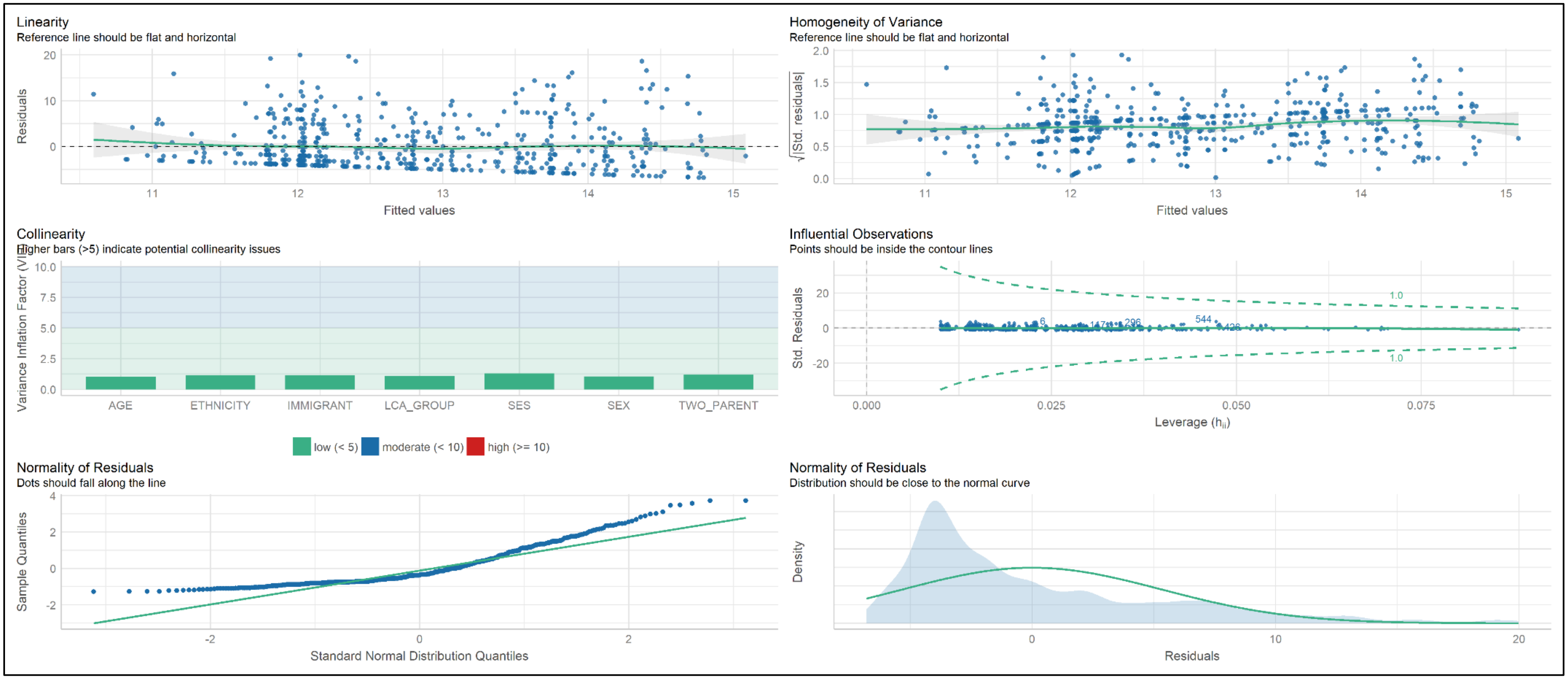

Missing covariate data were estimated using multiple imputation via chained equations. Data were analyzed with multivariable linear regression. Standard errors were corrected for using an HC3 adjustment. Analysis was run on 10 imputed data sets and the outcomes were combined using Rubin’s Rules. The first imputed data set was used to display distributional assumptions depicted above.

Figure S12. Distributional assumptions in the association between adversity and benevolence latent class and averaged older-and-younger-child anxiety, adjusted for age, sex, socioeconomic status, ethnicity, immigration status, and single parent status

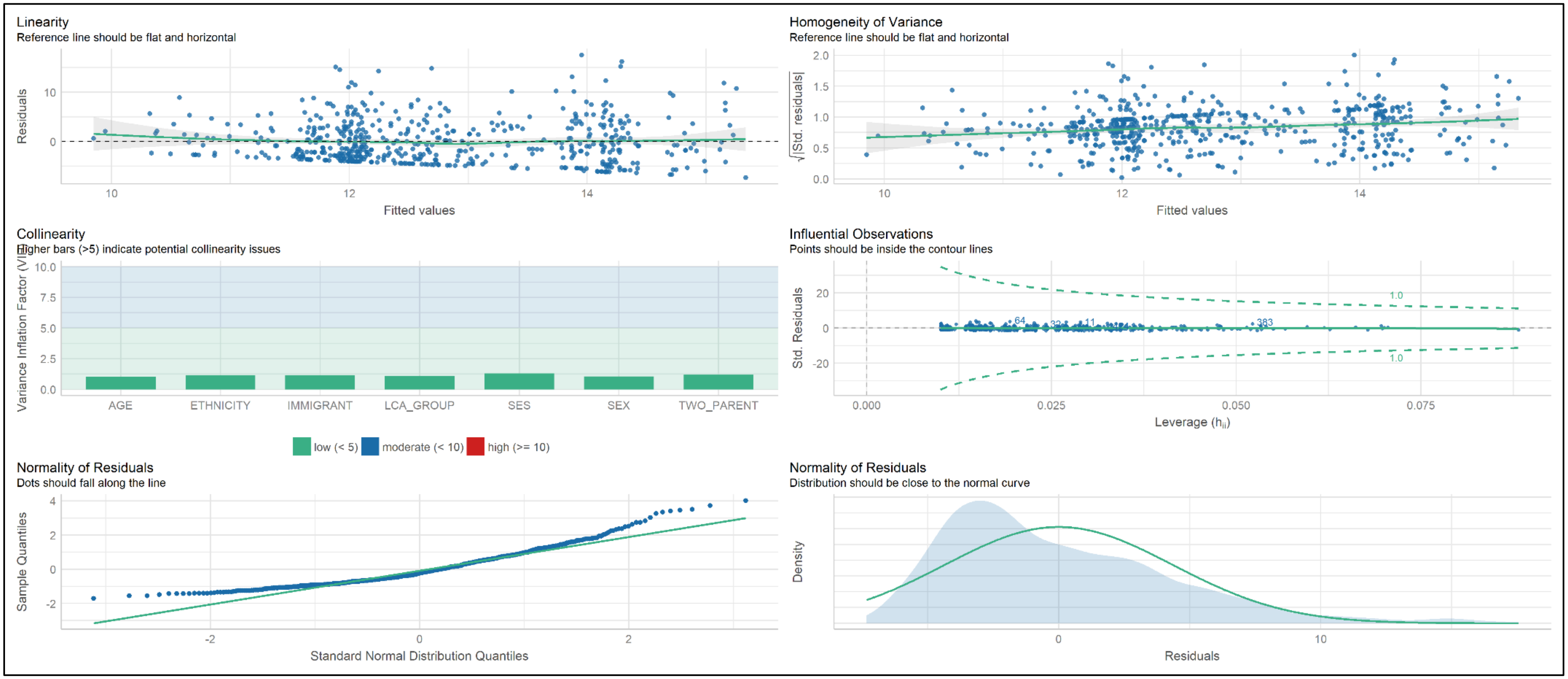

Missing covariate data were estimated using multiple imputation via chained equations. Data were analyzed with multivariable linear regression. Standard errors were corrected for using an HC3 adjustment. Analysis was run on 10 imputed data sets and the outcomes were combined using Rubin's Rules. The first imputed data set was used to display distributional assumptions depicted above.

Figure S13. Distributional assumptions in the association between adversity and benevolence latent class and older-born-child depression, adjusted for age, sex, socioeconomic status, ethnicity, immigration status, and single parent status

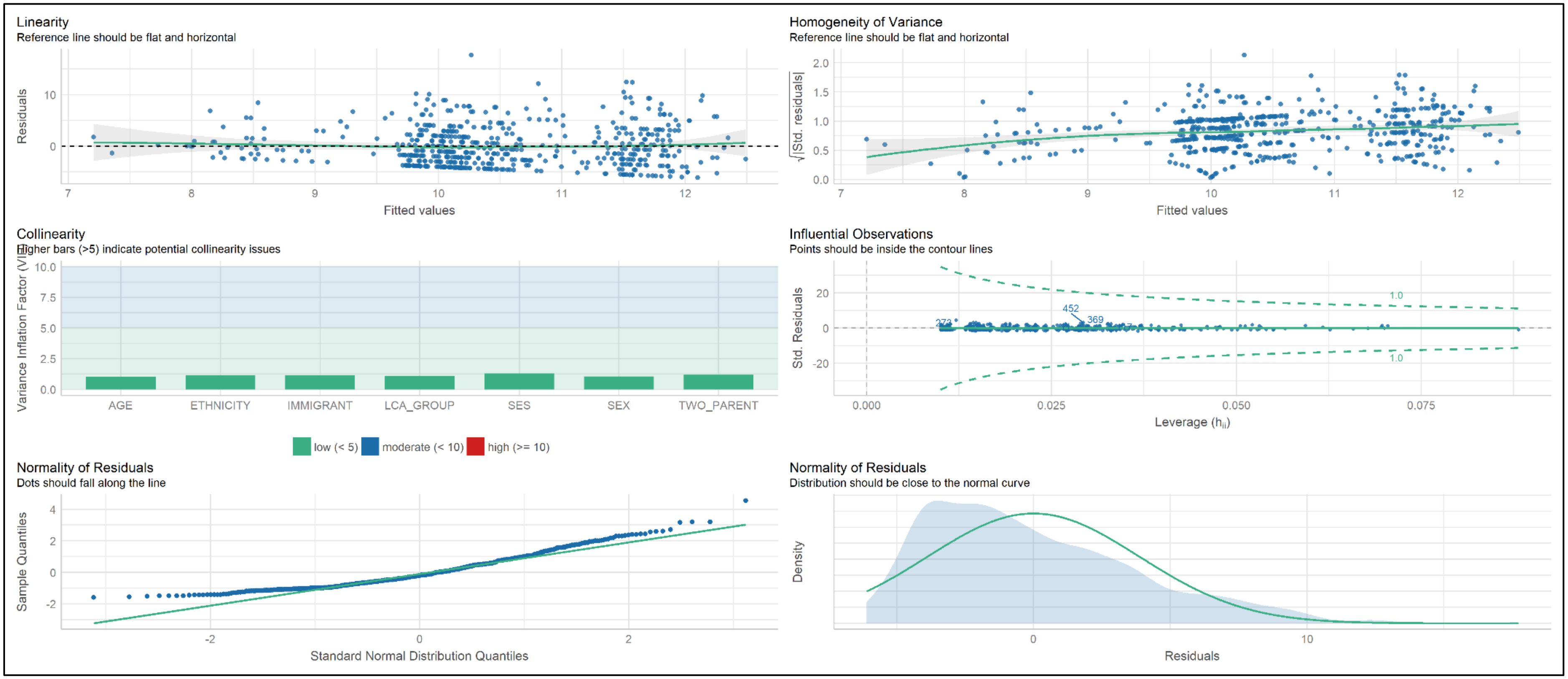

Missing covariate data were estimated using multiple imputation via chained equations. Data were analyzed with multivariable linear regression. Standard errors were corrected for using an HC3 adjustment. Analysis was run on 10 imputed data sets and the outcomes were combined using Rubin's Rules. The first imputed data set was used to display distributional assumptions depicted above.

Figure S14. Distributional assumptions in the association between adversity and benevolence latent class and younger-born-child depression, adjusted for age, sex, socioeconomic status, ethnicity, immigration status, and single parent status

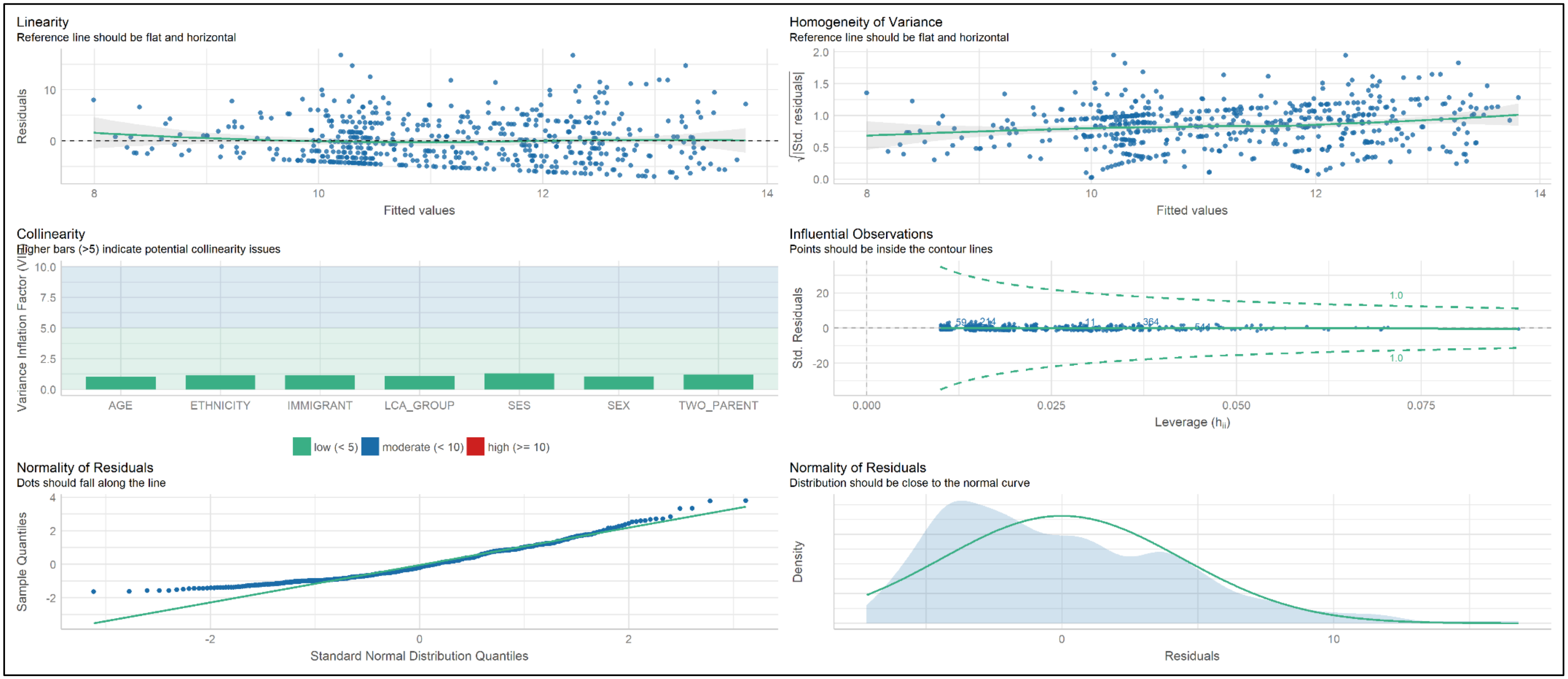

Missing covariate data were estimated using multiple imputation via chained equations. Data were analyzed with multivariable linear regression. Standard errors were corrected for using an HC3 adjustment. Analysis was run on 10 imputed data sets and the outcomes were combined using Rubin's Rules. The first imputed data set was used to display distributional assumptions depicted above.

Figure S15. Distributional assumptions in the association between adversity and benevolence latent class and averaged older-and-younger-child depression, adjusted for age, sex, socioeconomic status, ethnicity, immigration status, and single parent status

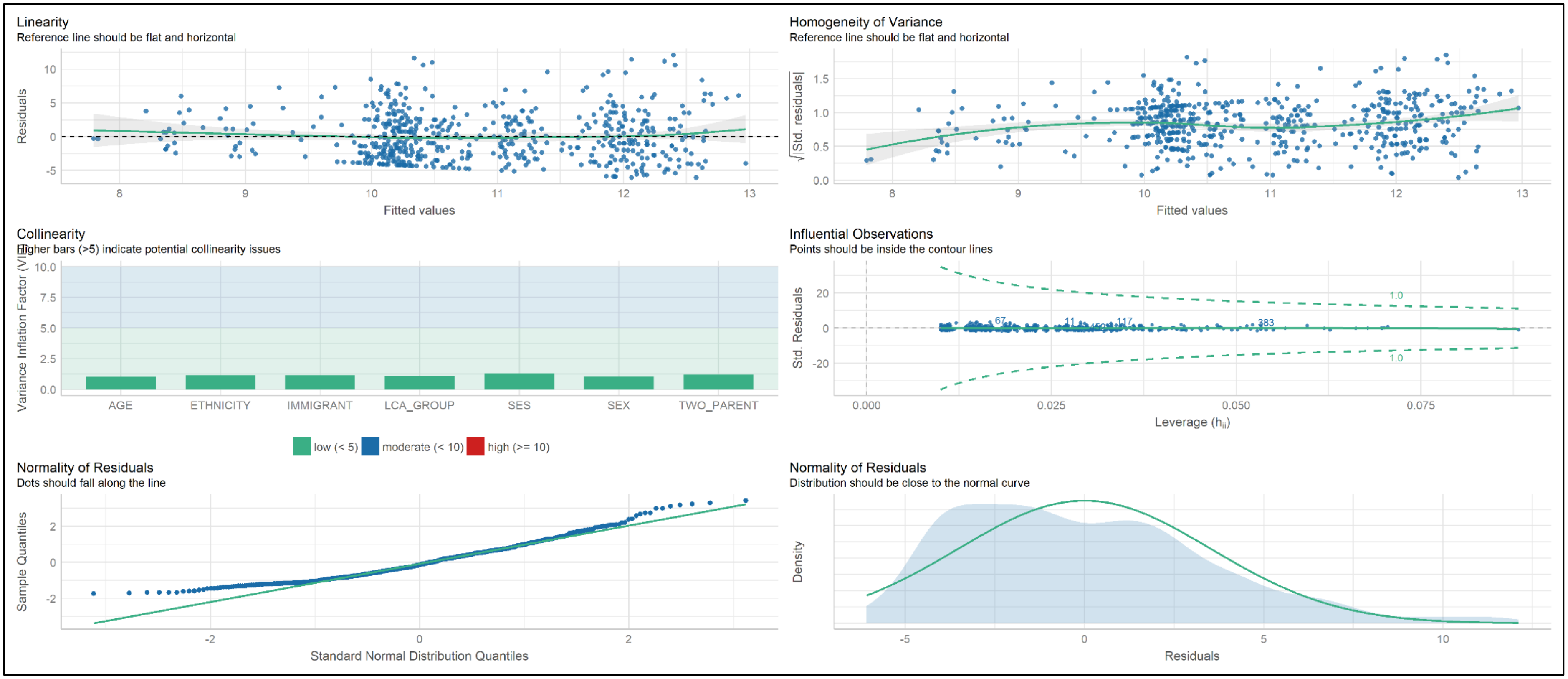

Missing covariate data were estimated using multiple imputation via chained equations. Data were analyzed with multivariable linear regression. Standard errors were corrected for using an HC3 adjustment. Analysis was run on 10 imputed data sets and the outcomes were combined using Rubin's Rules. The first imputed data set was used to display distributional assumptions depicted above.

Figure S16. Distributional assumptions in the association between adversity and benevolence latent class and older-born-child positive coping, adjusted for age, sex, socioeconomic status, ethnicity, immigration status, and single parent status

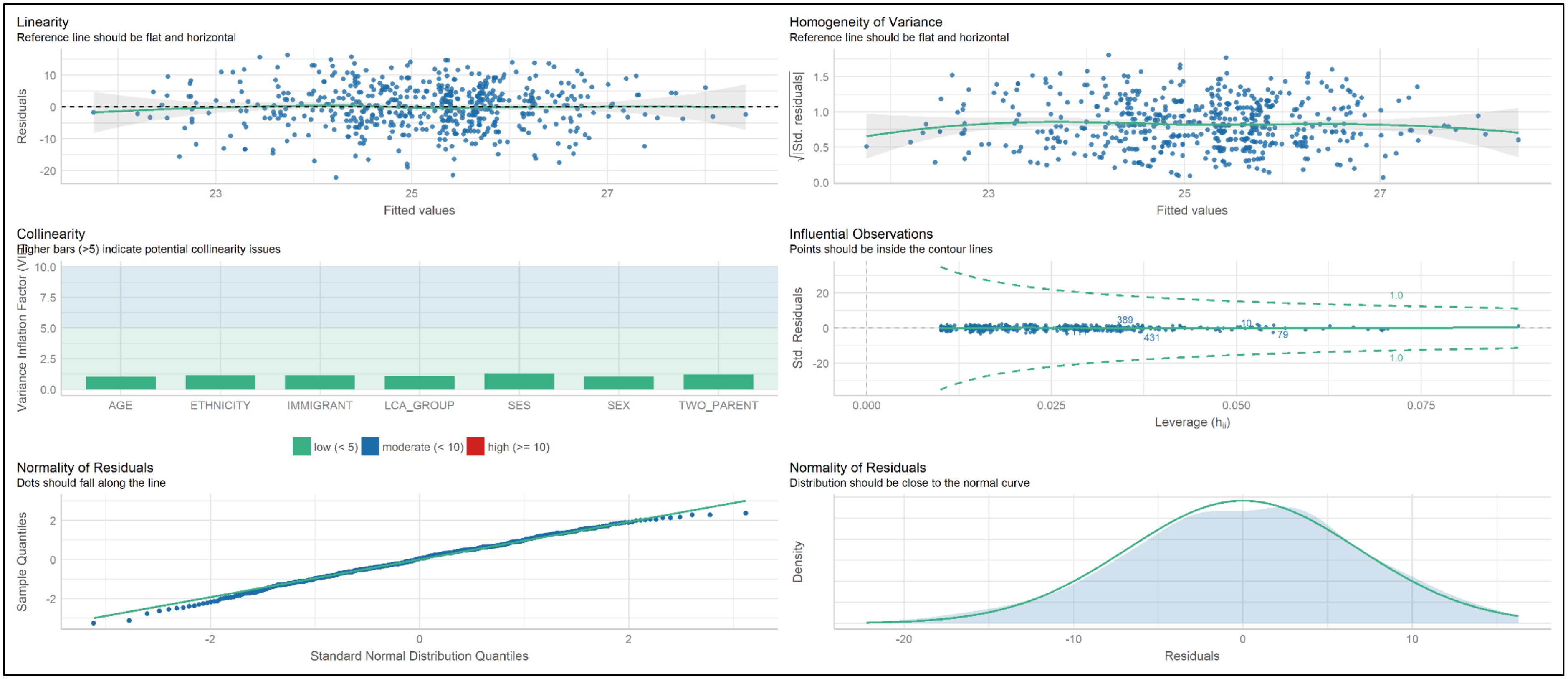

Missing covariate data were estimated using multiple imputation via chained equations. Data were analyzed with multivariable linear regression. Standard errors were corrected for using an HC3 adjustment. Analysis was run on 10 imputed data sets and the outcomes were combined using Rubin's Rules. The first imputed data set was used to display distributional assumptions depicted above.

Figure S17. Distributional assumptions in the association between adversity and benevolence latent class and younger-born-child positive coping, adjusted for age, sex, socioeconomic status, ethnicity, immigration status, and single parent status

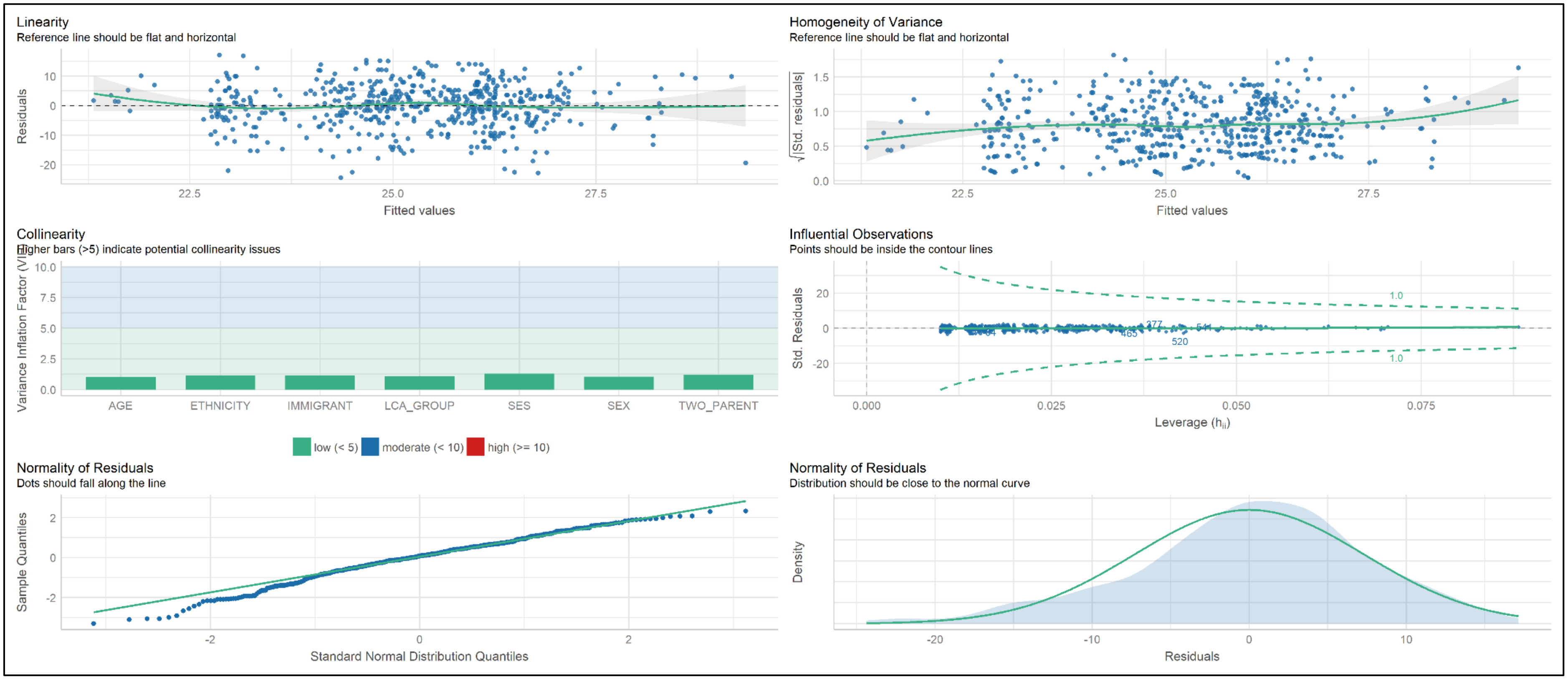

Missing covariate data were estimated using multiple imputation via chained equations. Data were analyzed with multivariable linear regression. Standard errors were corrected for using an HC3 adjustment. Analysis was run on 10 imputed data sets and the outcomes were combined using Rubin's Rules. The first imputed data set was used to display distributional assumptions depicted above.

Figure S18. Distributional assumptions in the association between adversity and benevolence latent class and averaged older-and-younger-child positive coping, adjusted for age, sex, socioeconomic status, ethnicity, immigration status, and single parent status

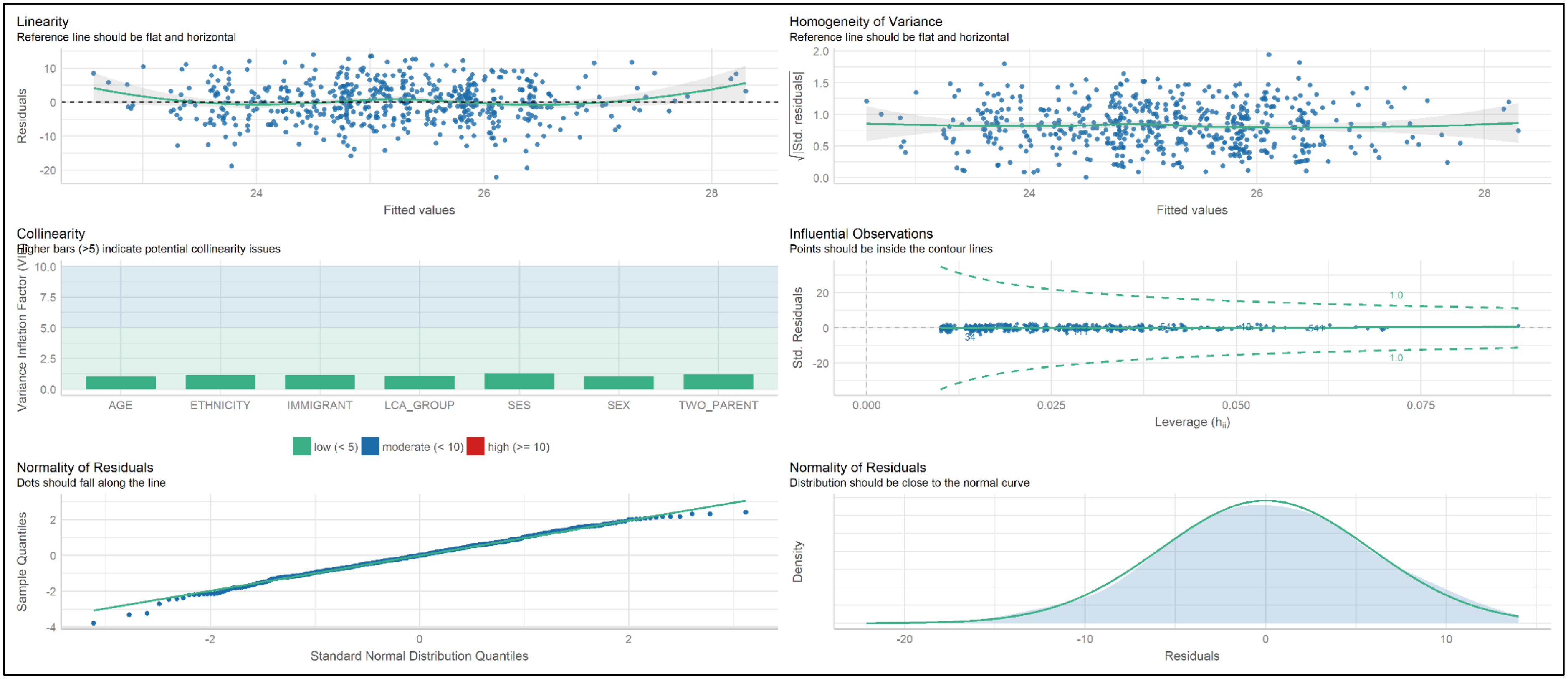

Missing covariate data were estimated using multiple imputation via chained equations. Data were analyzed with multivariable linear regression. Standard errors were corrected for using an HC3 adjustment. Analysis was run on 10 imputed data sets and the outcomes were combined using Rubin's Rules. The first imputed data set was used to display distributional assumptions depicted above.

Figure S19. Distributional assumptions in the association between adversity and benevolence latent class and older-born-child reported parenting quality, adjusted for age, sex, socioeconomic status, ethnicity, immigration status, and single parent status

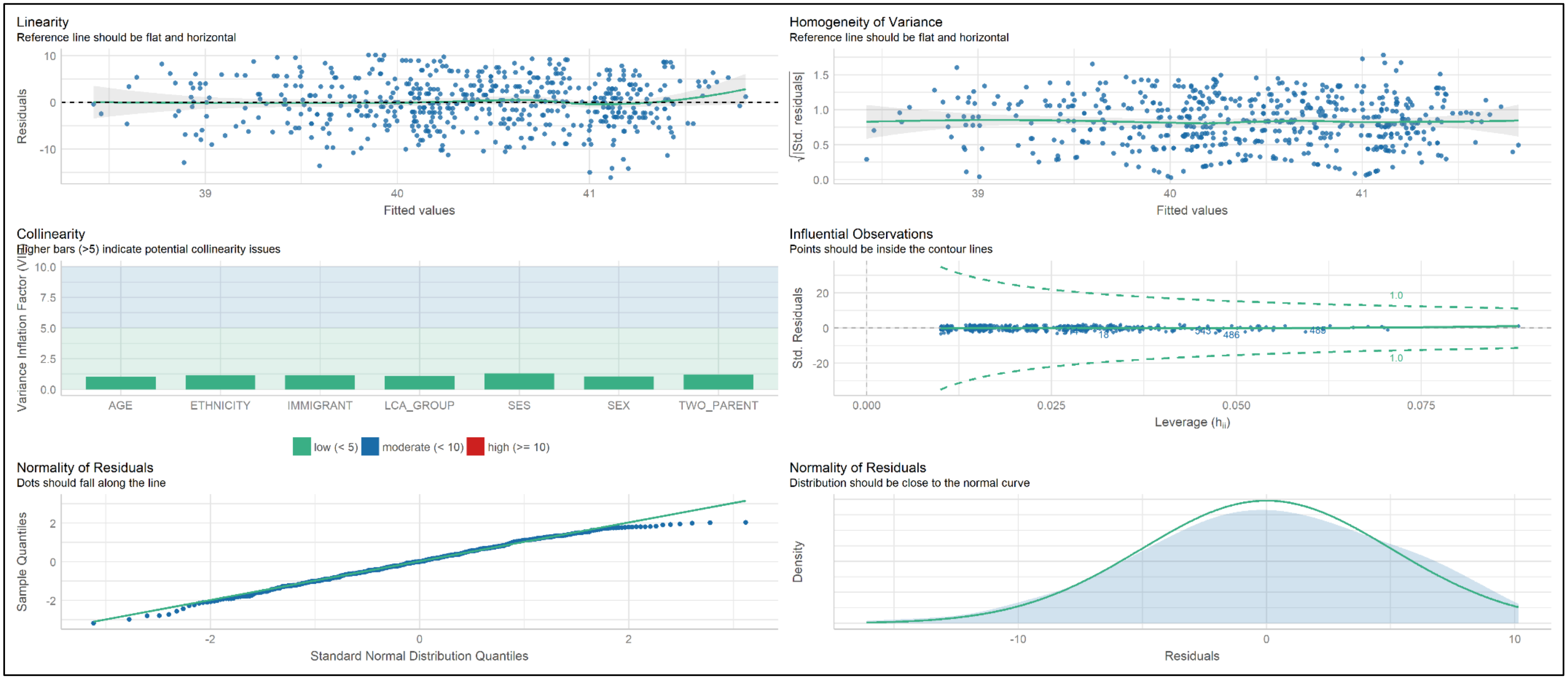

Missing covariate data were estimated using multiple imputation via chained equations. Data were analyzed with multivariable linear regression. Standard errors were corrected for using an HC3 adjustment. Analysis was run on 10 imputed data sets and the outcomes were combined using Rubin's Rules. The first imputed data set was used to display distributional assumptions depicted above.

Figure S20. Distributional assumptions in the association between adversity and benevolence latent class and younger-born-child reported parenting quality, adjusted for age, sex, socioeconomic status, ethnicity, immigration status, and single parent status

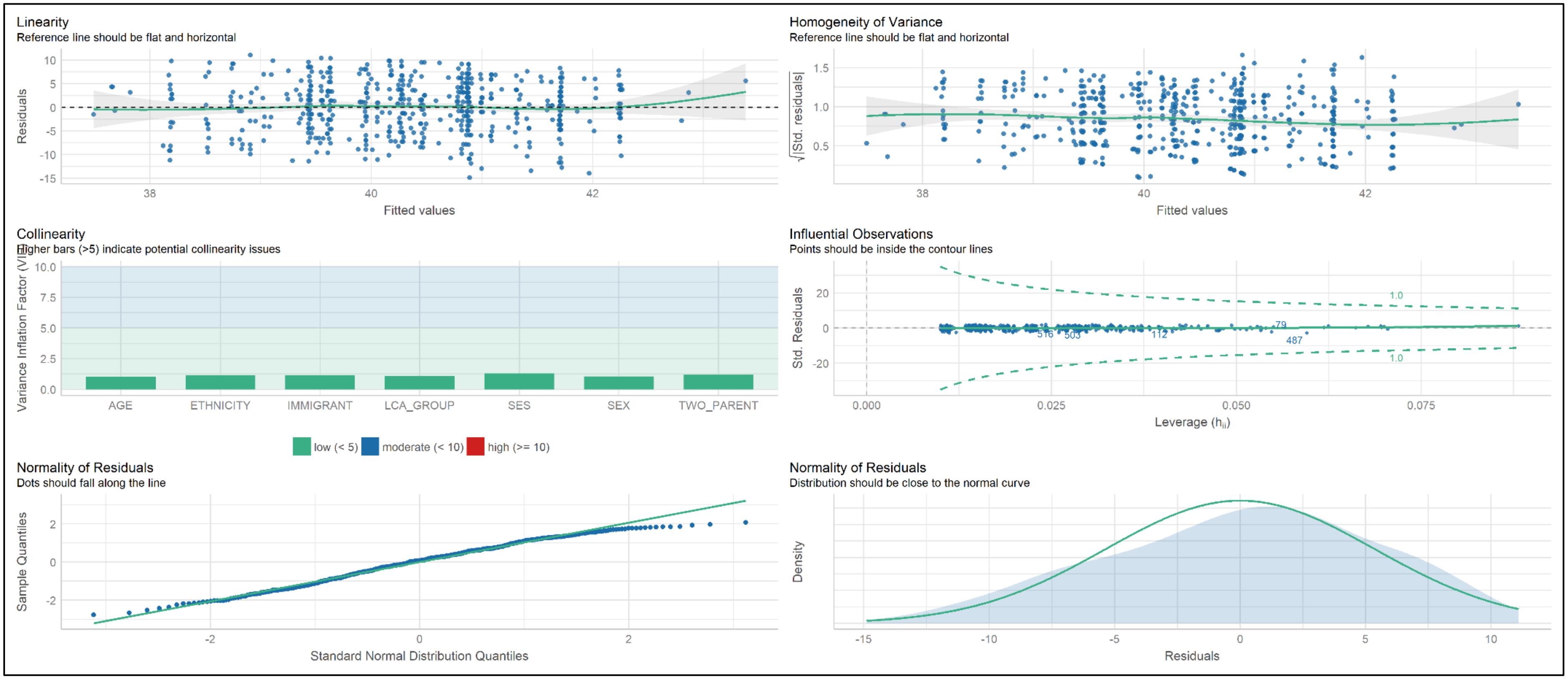

Missing covariate data were estimated using multiple imputation via chained equations. Data were analyzed with multivariable linear regression. Standard errors were corrected for using an HC3 adjustment. Analysis was run on 10 imputed data sets and the outcomes were combined using Rubin's Rules. The first imputed data set was used to display distributional assumptions depicted above.

Figure S21. Distributional assumptions in the association between adversity and benevolence latent class and averaged older-and-younger-child reported parenting quality, adjusted for age, sex, socioeconomic status, ethnicity, immigration status, and single parent status

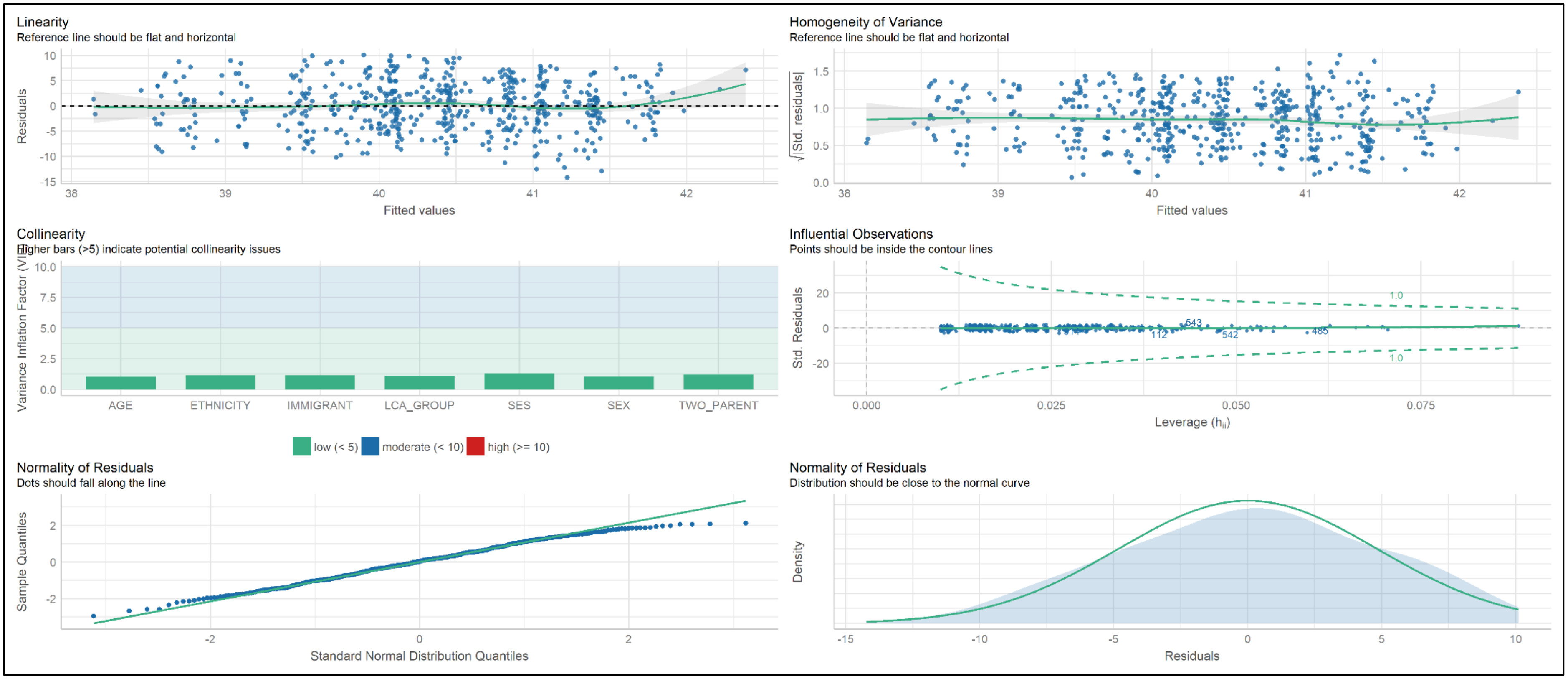

Missing covariate data were estimated using multiple imputation via chained equations. Data were analyzed with multivariable linear regression. Standard errors were corrected for using an HC3 adjustment. Analysis was run on 10 imputed data sets and the outcomes were combined using Rubin’s Rules. The first imputed data set was used to display distributional assumptions depicted above.

Figure S22. Correlational matrix for older-born-child outcomes of anger, anxiety, depression, positive coping, and parenting quality

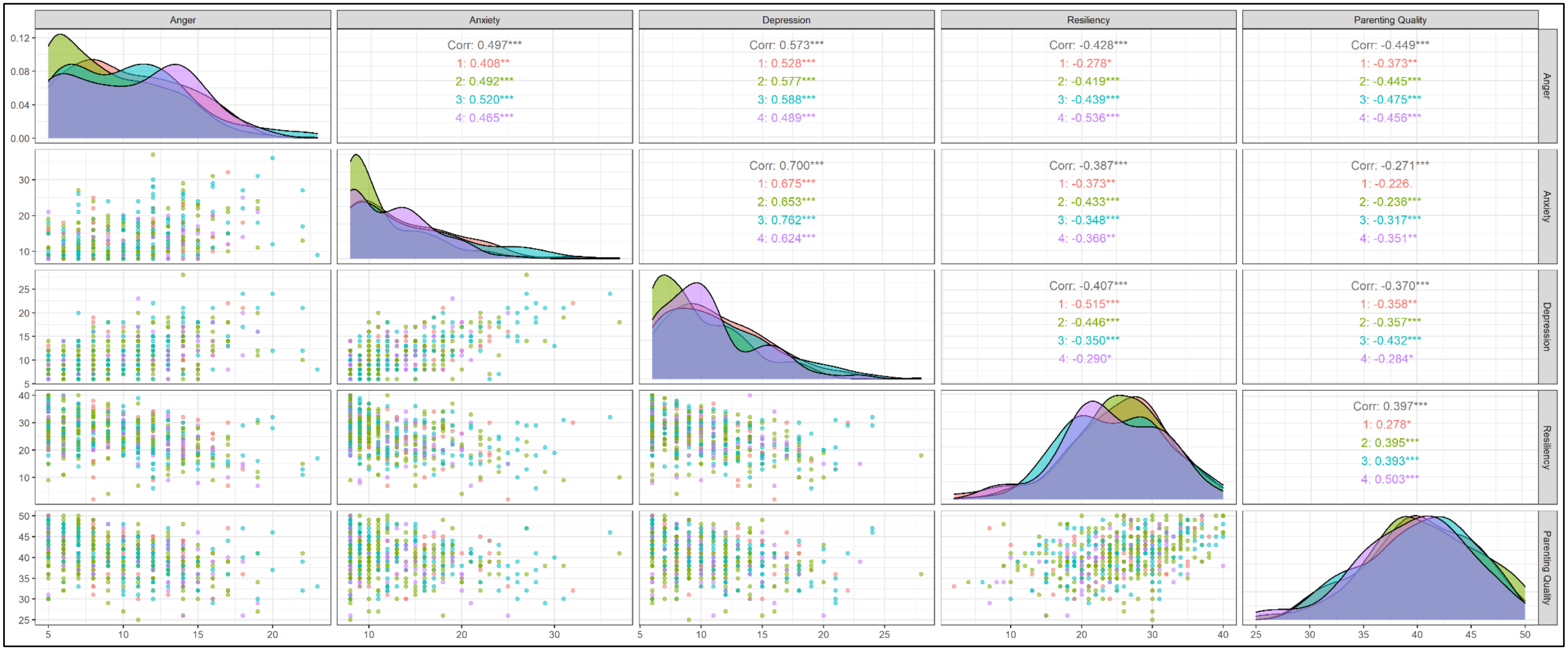

Figure S23. Correlational matrix for younger-born-child outcomes of anger, anxiety, depression, positive coping, and parenting quality

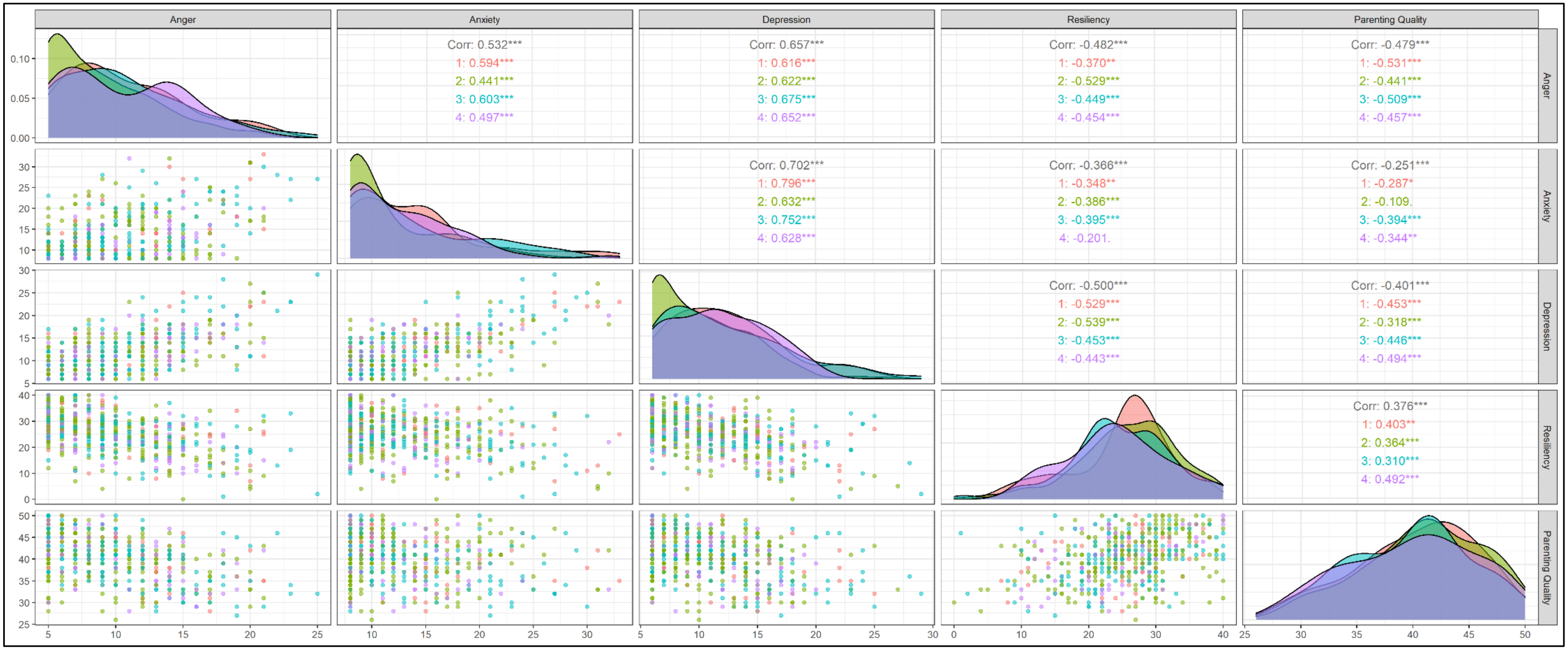

Figure S24. Correlational matrix for averaged older-and-younger-child outcomes of anger, anxiety, depression, positive coping, and parenting quality

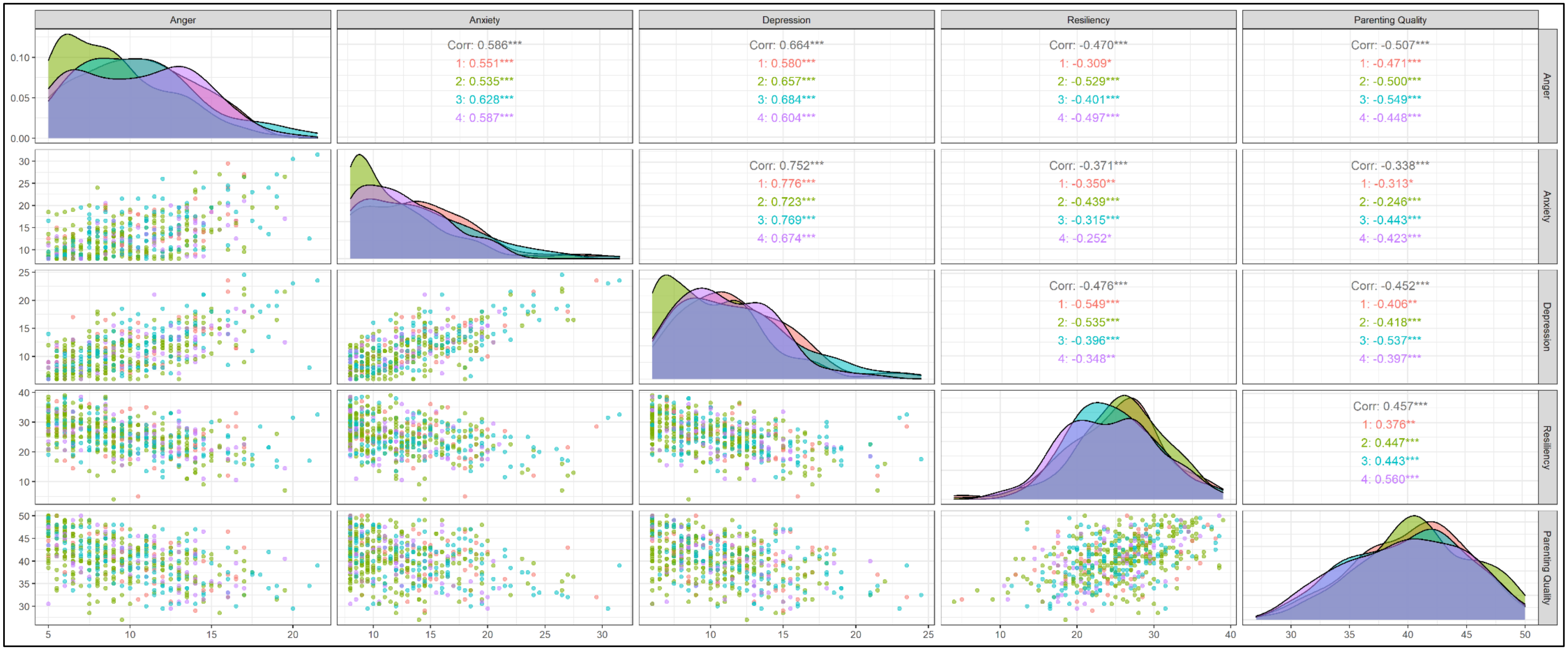

Table S8. Distribution of younger child mental health, positive coping, and parenting quality, among latent classes of adversity and benevolence.

	Low ACE/High BCE	Mod ACE/High BCE	Mod ACE/Low BCE	High ACE/Mod BCE	p-value
**Younger Child Anger (Mean, SD)**	8.96 (3.91)	10.53 (4.34)	10.74 (4.59)	10.75 (4.38)	*F=7.704*, p<0.001
MISSING	1	0	0	0	
**Younger Child Anxiety (Mean, SD)**	11.97 (4.98)	12.71 (4.80)	13.66 (5.95)	14.26 (6.15)	*F=4.867*, p=0.002
MISSING	1	0	0	0	
**Younger Child Depression (Mean, SD)**	10.25 (4.06)	11.76 (4.05)	12.23 (5.25)	12.33 (4.63)	*F=8.312*, p<0.001
MISSING	1	0	0	0	
**Younger Child Positive Coping (Mean, SD)**	25.93 (7.41)	24.21 (7.56)	24.64 (7.54)	24.98 (7.27)	*F=1.545*, p=0.202
MISSING	1	0	0	0	
**Younger Child Parenting Quality (Mean, SD)**	40.88 (5.39)	39.91 (5.75)	39.70 (5.45)	40.36 (5.27)	*F=1.708*, p=0.164
MISSING	1	0	0	0	

p-value represents results from a t-test (continuous variables) or chi-square test (categorical variables)

Table S9. Multiple linear regression model in the association between adversity and benevolence latent class and older-born-child anger (n=545)

Pairwise Comparison		Unadjusted			Adjusted†		
Reference	Comparator	Mean Difference	Standard Error	t-statistic	Mean Difference	Standard Error	t-statistic
High ACE/Mod BCE	Low ACE/High BCE	-1.06	0.53	-1.99*	-0.96	0.55	-1.74
High ACE/Mod BCE	Mod ACE/High BCE	0.18	0.59	0.31	0.32	0.60	0.52
High ACE/Mod BCE	Mod ACE/Low BCE	0.33	0.69	0.47	0.32	0.70	0.46
Low ACE/High BCE	Mod ACE/High BCE	1.24	0.41	3.04**	1.28	0.41	3.10**
Low ACE/High BCE	Mod ACE/Low BCE	1.38	0.54	2.57*	1.28	0.55	2.35*
Mod ACE/High BCE	Mod ACE/Low BCE	0.14	0.60	0.24	0.01	0.60	0.01

†Adjusted for age, sex, socioeconomic status, ethnicity, immigration status, and one-parent household status
*p<0.05
**p<0.01
***p<0.001

Table S10. Multiple linear regression model in the association between adversity and benevolence latent class and younger-born-child anger (n=546)

Pairwise Comparison		Unadjusted			Adjusted†		
Reference	Comparator	Mean Difference	Standard Error	t-statistic	Mean Difference	Standard Error	t-statistic
High ACE/Mod BCE	Low ACE/High BCE	-1.79	0.61	-2.92**	-1.53	0.62	-2.45*
High ACE/Mod BCE	Mod ACE/High BCE	-0.01	0.68	-0.02	0.30	0.69	0.44
High ACE/Mod BCE	Mod ACE/Low BCE	-0.23	0.77	-0.29	-0.28	0.77	-0.37
Low ACE/High BCE	Mod ACE/High BCE	1.78	0.45	3.95***	1.83	0.47	3.92***
Low ACE/High BCE	Mod ACE/Low BCE	1.57	0.57	2.73**	1.25	0.59	2.13*
Mod ACE/High BCE	Mod ACE/Low BCE	-0.21	0.65	-0.33	-0.58	0.67	-0.88

†Adjusted for age, sex, socioeconomic status, ethnicity, immigration status, and one-parent household status
*p<0.05
**p<0.01
***p<0.001

Table S11. Multiple linear regression model in the association between adversity and benevolence latent class and averaged older and younger child anger (n=545)

Pairwise Comparison		Unadjusted			Adjusted†		
Reference	Comparator	Mean Difference	Standard Error	t-statistic	Mean Difference	Standard Error	t-statistic
High ACE/Mod BCE	Low ACE/High BCE	-1.42	0.48	-2.97**	-1.25	0.50	-2.51*
High ACE/Mod BCE	Mod ACE/High BCE	0.10	0.53	0.18	0.32	0.55	0.59
High ACE/Mod BCE	Mod ACE/Low BCE	0.02	0.62	0.04	0.00	0.63	0.00
Low ACE/High BCE	Mod ACE/High BCE	1.52	0.37	4.13***	1.57	0.37	4.20***
Low ACE/High BCE	Mod ACE/Low BCE	1.45	0.49	2.95**	1.25	0.50	2.51*
Mod ACE/High BCE	Mod ACE/Low BCE	-0.07	0.54	-0.13	-0.32	0.55	-0.59

†Adjusted for age, sex, socioeconomic status, ethnicity, immigration status, and one-parent household status
*p<0.05
**p<0.01
***p<0.001

Table S12. Multiple linear regression model in the association between adversity and benevolence latent class and older-child anxiety (n=545)

Pairwise Comparison		Unadjusted			Adjusted†		
Reference	Comparator	Mean Difference	Standard Error	t-statistic	Mean Difference	Standard Error	t-statistic
High ACE/Mod BCE	Low ACE/High BCE	-1.89	0.77	-2.46*	-2.02	0.81	-2.49*
High ACE/Mod BCE	Mod ACE/High BCE	0.69	0.89	0.78	0.62	0.92	0.67
High ACE/Mod BCE	Mod ACE/Low BCE	-1.25	0.88	-1.41	-1.30	0.91	-1.43
Low ACE/High BCE	Mod ACE/High BCE	2.59	0.61	4.26***	2.64	0.61	4.32***
Low ACE/High BCE	Mod ACE/Low BCE	0.65	0.60	1.08	0.73	0.62	1.17
Mod ACE/High BCE	Mod ACE/Low BCE	-1.94	0.75	-2.60**	-1.91	0.77	-2.50*

†Adjusted for age, sex, socioeconomic status, ethnicity, immigration status, and one-parent household status
*p<0.05
**p<0.01
***p<0.001

Table S13. Multiple linear regression model in the association between adversity and benevolence latent class and younger-born-child anxiety (n=546)

Pairwise Comparison		Unadjusted			Adjusted†		
Reference	Comparator	Mean Difference	Standard Error	t-statistic	Mean Difference	Standard Error	t-statistic
High ACE/Mod BCE	Low ACE/High BCE	-2.29	0.85	-2.69**	-2.35	0.87	-2.69**
High ACE/Mod BCE	Mod ACE/High BCE	-0.60	0.94	-0.65	-0.68	0.94	-0.72
High ACE/Mod BCE	Mod ACE/Low BCE	-1.55	0.98	-1.58	-1.60	1.00	-1.60
Low ACE/High BCE	Mod ACE/High BCE	1.68	0.58	2.90**	1.67	0.60	2.80**
Low ACE/High BCE	Mod ACE/Low BCE	0.74	0.65	1.13	0.75	0.68	1.09
Mod ACE/High BCE	Mod ACE/Low BCE	-0.94	0.76	-1.24	-0.92	0.79	-1.16

†Adjusted for age, sex, socioeconomic status, ethnicity, immigration status, and one-parent household status
*p<0.05
**p<0.01
***p<0.001

Table S14. Multiple linear regression model in the association between adversity and benevolence latent class and averaged older and younger child anxiety (n=545)

Pairwise Comparison		Unadjusted			Adjusted†		
Reference	Comparator	Mean Difference	Standard Error	t-statistic	Mean Difference	Standard Error	t-statistic
High ACE/Mod BCE	Low ACE/High BCE	-2.08	0.67	-3.13**	-2.19	0.69	-3.16**
High ACE/Mod BCE	Mod ACE/High BCE	0.06	0.75	0.08	-0.01	0.76	-0.01
High ACE/Mod BCE	Mod ACE/Low BCE	-1.41	0.76	-1.85	-1.48	0.78	-1.90
Low ACE/High BCE	Mod ACE/High BCE	2.14	0.49	4.36***	2.18	0.49	4.40***
Low ACE/High BCE	Mod ACE/Low BCE	0.67	0.51	1.31	0.71	0.53	1.34
Mod ACE/High BCE	Mod ACE/Low BCE	-1.47	0.62	-2.39*	-1.47	0.63	-2.32**

†Adjusted for age, sex, socioeconomic status, ethnicity, immigration status, and one-parent household status
*p<0.05
**p<0.01
***p<0.001

Table S15. Multiple linear regression model in the association between adversity and benevolence latent class and older-child depression (n=545)

Pairwise Comparison		Unadjusted			Adjusted†		
Reference	Comparator	Mean Difference	Standard Error	t-statistic	Mean Difference	Standard Error	t-statistic
High ACE/Mod BCE	Low ACE/High BCE	-1.49	0.56	-2.66**	-1.48	0.57	-2.57*
High ACE/Mod BCE	Mod ACE/High BCE	0.09	0.63	0.14	0.17	0.64	0.26
High ACE/Mod BCE	Mod ACE/Low BCE	-0.78	0.69	-1.13	-0.82	0.70	-1.18
Low ACE/High BCE	Mod ACE/High BCE	1.57	0.43	3.67***	1.64	0.44	3.76***
Low ACE/High BCE	Mod ACE/Low BCE	0.71	0.51	1.39	0.66	0.54	1.22
Mod ACE/High BCE	Mod ACE/Low BCE	-0.86	0.59	-1.47	-0.99	0.61	-1.62

†Adjusted for age, sex, socioeconomic status, ethnicity, immigration status, and one-parent household status
*p<0.05
**p<0.01
***p<0.001

Table S16. Multiple linear regression model in the association between adversity and benevolence latent class and younger-child depression (n=546)

Pairwise Comparison		Unadjusted			Adjusted†		
Reference	Comparator	Mean Difference	Standard Error	t-statistic	Mean Difference	Standard Error	t-statistic
High ACE/Mod BCE	Low ACE/High BCE	-2.08	0.65	-3.22**	-2.08	0.67	-3.11**
High ACE/Mod BCE	Mod ACE/High BCE	-0.10	0.74	-0.14	-0.06	0.75	-0.07
High ACE/Mod BCE	Mod ACE/Low BCE	-0.57	0.77	-0.74	-0.60	0.78	-0.77
Low ACE/High BCE	Mod ACE/High BCE	1.98	0.50	3.95***	2.02	0.51	3.98***
Low ACE/High BCE	Mod ACE/Low BCE	1.51	0.55	2.77**	1.48	0.57	2.61**
Mod ACE/High BCE	Mod ACE/Low BCE	-0.47	0.65	-0.72	-0.54	0.67	-0.80

†Adjusted for age, sex, socioeconomic status, ethnicity, immigration status, and one-parent household status
*p<0.05
**p<0.01
***p<0.001

Table S17. Multiple linear regression model in the association between adversity and benevolence latent class and averaged older and younger child depression (n=545)

Pairwise Comparison		Unadjusted			Adjusted†		
Reference	Comparator	Mean Difference	Standard Error	t-statistic	Mean Difference	Standard Error	t-statistic
High ACE/Mod BCE	Low ACE/High BCE	-1.78	0.52	-3.43***	-1.78	0.54	-3.31**
High ACE/Mod BCE	Mod ACE/High BCE	0.01	0.58	0.03	0.07	0.59	0.13
High ACE/Mod BCE	Mod ACE/Low BCE	-0.71	0.62	-1.15	-0.74	0.63	-1.18
Low ACE/High BCE	Mod ACE/High BCE	1.79	0.40	4.53***	1.85	0.40	4.67***
Low ACE/High BCE	Mod ACE/Low BCE	1.07	0.45	2.38*	1.04	0.47	2.20*
Mod ACE/High BCE	Mod ACE/Low BCE	-0.73	0.52	-1.39	-0.82	0.54	-1.52

†Adjusted for age, sex, socioeconomic status, ethnicity, immigration status, and one-parent household status
*p<0.05
**p<0.01
***p<0.001

Table S18. Multiple linear regression model in the association between adversity and benevolence latent class and older-child positive coping (n=545)

Pairwise Comparison		Unadjusted			Adjusted†		
Reference	Comparator	Mean Difference	Standard Error	t-statistic	Mean Difference	Standard Error	t-statistic
High ACE/Mod BCE	Low ACE/High BCE	0.60	1.04	0.57	0.37	1.04	0.36
High ACE/Mod BCE	Mod ACE/High BCE	-0.26	1.12	-0.23	-0.46	1.11	-0.42
High ACE/Mod BCE	Mod ACE/Low BCE	-0.68	1.28	-0.53	-0.62	1.28	-0.48
Low ACE/High BCE	Mod ACE/High BCE	-0.85	0.72	-1.19	-0.83	0.75	-1.11
Low ACE/High BCE	Mod ACE/Low BCE	-1.28	0.95	-1.35	-0.99	0.95	-1.04
Mod ACE/High BCE	Mod ACE/Low BCE	-0.43	1.04	-0.41	-0.16	1.06	-0.15

†Adjusted for age, sex, socioeconomic status, ethnicity, immigration status, and one-parent household status
*p<0.05
**p<0.01
***p<0.001

Table S19. Multiple linear regression model in the association between adversity and benevolence latent class and younger-child positive coping (n=546)

Pairwise Comparison		Unadjusted			Adjusted†		
Reference	Comparator	Mean Difference	Standard Error	t-statistic	Mean Difference	Standard Error	t-statistic
High ACE/Mod BCE	Low ACE/High BCE	0.95	1.04	0.91	0.91	1.04	0.87
High ACE/Mod BCE	Mod ACE/High BCE	-0.34	1.13	-0.30	-0.39	1.11	-0.35
High ACE/Mod BCE	Mod ACE/Low BCE	-0.77	1.31	-0.59	-0.38	1.28	-0.29
Low ACE/High BCE	Mod ACE/High BCE	-1.29	0.77	-1.66	-1.30	0.81	-1.61
Low ACE/High BCE	Mod ACE/Low BCE	-1.72	1.02	-1.69	-1.29	1.03	-1.25
Mod ACE/High BCE	Mod ACE/Low BCE	-0.43	1.10	-0.39	0.01	1.13	0.01

†Adjusted for age, sex, socioeconomic status, ethnicity, immigration status, and one-parent household status
*p<0.05
**p<0.01
***p<0.001

Table S20. Multiple linear regression model in the association between adversity and benevolence latent class and averaged older and younger child positive coping (n=545)

Pairwise Comparison		Unadjusted			Adjusted†		
Reference	Comparator	Mean Difference	Standard Error	t-statistic	Mean Difference	Standard Error	t-statistic
High ACE/Mod BCE	Low ACE/High BCE	0.76	0.89	0.86	0.63	0.88	0.72
High ACE/Mod BCE	Mod ACE/High BCE	-0.28	0.93	-0.31	-0.41	0.92	-0.45
High ACE/Mod BCE	Mod ACE/Low BCE	-0.73	1.10	-0.67	-0.51	1.09	-0.47
Low ACE/High BCE	Mod ACE/High BCE	-1.05	0.59	-1.77	-1.05	0.62	-1.70
Low ACE/High BCE	Mod ACE/Low BCE	-1.49	0.83	-1.81	-1.15	0.84	-1.37
Mod ACE/High BCE	Mod ACE/Low BCE	-0.45	0.88	-0.51	-0.10	0.90	-0.11

†Adjusted for age, sex, socioeconomic status, ethnicity, immigration status, and one-parent household status
*p<0.05
**p<0.01
***p<0.001

Table S21. Multiple linear regression model in the association between adversity and benevolence latent class and older-child parenting quality (n=545)

Pairwise Comparison		Unadjusted			Adjusted†		
Reference	Comparator	Mean Difference	Standard Error	t-statistic	Mean Difference	Standard Error	t-statistic
High ACE/Mod BCE	Low ACE/High BCE	0.46	0.71	0.64	0.55	0.73	0.76
High ACE/Mod BCE	Mod ACE/High BCE	-0.04	0.76	-0.05	-0.03	0.78	-0.03
High ACE/Mod BCE	Mod ACE/Low BCE	-0.51	0.89	-0.57	-0.52	0.90	-0.57
Low ACE/High BCE	Mod ACE/High BCE	-0.50	0.52	-0.96	-0.58	0.54	-1.07
Low ACE/High BCE	Mod ACE/Low BCE	-0.96	0.70	-1.39	-1.07	0.72	-1.47
Mod ACE/High BCE	Mod ACE/Low BCE	-0.47	0.75	-0.63	-0.49	0.78	-0.63

†Adjusted for age, sex, socioeconomic status, ethnicity, immigration status, and one-parent household status
*p<0.05
**p<0.01
***p<0.001

Table S22. Multiple linear regression model in the association between adversity and benevolence latent class and younger-child parenting quality (n=546)

Pairwise Comparison		Unadjusted			Adjusted†		
Reference	Comparator	Mean Difference	Standard Error	t-statistic	Mean Difference	Standard Error	t-statistic
High ACE/Mod BCE	Low ACE/High BCE	0.52	0.76	0.69	0.59	0.76	0.78
High ACE/Mod BCE	Mod ACE/High BCE	-0.66	0.82	-0.81	-0.61	0.83	-0.74
High ACE/Mod BCE	Mod ACE/Low BCE	-0.45	0.97	-0.46	-0.34	0.97	-0.35
Low ACE/High BCE	Mod ACE/High BCE	-1.19	0.56	-2.12**	-1.20	0.57	-2.11**
Low ACE/High BCE	Mod ACE/Low BCE	-0.97	0.77	-1.27	-0.94	0.78	-1.21
Mod ACE/High BCE	Mod ACE/Low BCE	0.22	0.83	0.26	0.27	0.84	0.32

†Adjusted for age, sex, socioeconomic status, ethnicity, immigration status, and one-parent household status
*p<0.05
**p<0.01
***p<0.001

Table S23. Multiple linear regression model in the association between adversity and benevolence latent class and averaged older and younger child parenting quality (n=545)

Pairwise Comparison		Unadjusted			Adjusted†		
Reference	Comparator	Mean Difference	Standard Error	t-statistic	Mean Difference	Standard Error	t-statistic
High ACE/Mod BCE	Low ACE/High BCE	0.49	0.67	0.73	0.57	0.68	0.84
High ACE/Mod BCE	Mod ACE/High BCE	-0.36	0.73	-0.49	-0.32	0.74	-0.43
High ACE/Mod BCE	Mod ACE/Low BCE	-0.43	0.86	-0.50	-0.39	0.87	-0.45
Low ACE/High BCE	Mod ACE/High BCE	-0.84	0.51	-1.66	-0.90	0.52	-1.71
Low ACE/High BCE	Mod ACE/Low BCE	-0.92	0.69	-1.35	-0.96	0.70	-1.37
Mod ACE/High BCE	Mod ACE/Low BCE	-0.08	0.74	-0.11	-0.07	0.76	-0.09

†Adjusted for age, sex, socioeconomic status, ethnicity, immigration status, and one-parent household status

*p<0.05

**p<0.01

***p<0.001

Table S24. Frequency of missing data per variable

Variable	Count	Method for Addressing Missing Data
Latent Class Analysis Indicators		
Complete missingness on all indicator items of ACEs and BCEs	2	Listwise deletion
Partial missingness on some indicator items of ACEs and BCEs	5	Full Information Maximum Likelihood Estimation
Outcome Variables		
Child (older) Anger	2	Excluded from child anger analysis
Child (older) Anxiety	2	Excluded from child anxiety analysis
Child (older) Depression	2	Excluded from child depression analysis
Child (older) Positive Coping	2	Excluded from child positive coping analysis
Child (older) Parenting quality	2	Excluded from child parenting quality analysis
Child (younger) Anger	1	Excluded from child anger analysis
Child (younger) Anxiety	1	Excluded from child anxiety analysis
Child (younger) Depression	1	Excluded from child depression analysis
Child (younger) Positive Coping	1	Excluded from child positive coping analysis
Child (younger) Parenting quality	1	Excluded from child parenting quality analysis
Parent Anxiety	2	Excluded from anxiety analysis
Parent Psychological Distress	1	Excluded from distress analysis
Parent Posttraumatic stress	2	Excluded from posttraumatic stress analysis
Parent Substance use	2	Excluded from substance use analysis
Parent Family Dysfunction	0	Excluded from family dysfunction analysis
Covariates		
Parent Age	22	Multiple imputation via chained equation on 10 imputed data sets, combined using Rubin's Rules
Parent Sex	19	
Parent Socioeconomic status	1	
Parent Ethnicity	80	
Parent Immigration status	3	
One-parent household status	0	

Table S25. R Packages used for analyses and data presentation in R Studio (version 4.0.2)

Package Name	Version
**car**	3.0-10
**devtools**	2.3.2
**GGally**	2.1.2
**ggdist**	3.0.1
**ggplot2**	3.3.5
**kableExtra**	1.3.4
**lmtest**	0.9-39
**mice**	3.0-10
**patchwork**	3.14.0
**performance**	0.8.0
**sandwich**	3.0-1
**see**	0.6.8
**tidyverse**	1.3.1
**zoo**	1.8-8
